# Mesenchymal GDNF promotes intestinal enterochromaffin cell differentiation

**DOI:** 10.1016/j.isci.2024.111246

**Published:** 2024-10-24

**Authors:** Toni T. Lemmetyinen, Emma W. Viitala, Linnea Wartiovaara, Pekka Päivinen, Heikki T. Virtanen, Nalle Pentinmikko, Pekka Katajisto, Tomi P. Mäkelä, Timothy C. Wang, Jaan-Olle Andressoo, Saara Ollila

**Affiliations:** 1 Translational Cancer Medicine Program, University of Helsinki, 00014 Helsinki, Finland; 2 HiLIFE-Helsinki Institute of Life Science, University of Helsinki, 00014 Helsinki, Finland; 3 iCAN Digital Precision Cancer Medicine Flagship, University of Helsinki, 00014 Helsinki, Finland; 4 Department of Pharmacology, Faculty of Medicine, Helsinki Institute of Life Science, University of Helsinki, 00290 Helsinki, Finland; 5 The Francis Crick Institute, London NW1 1AY, UK; 6 Institute of Biotechnology, HiLIFE, University of Helsinki, 00014 Helsinki, Finland; 7 Faculty of Biological and Environmental Sciences, Molecular and Integrative Biosciences Research Program, University of Helsinki, 00014 Helsinki, Finland; 8 Department of Cell and Molecular Biology, Karolinska Institutet, 17177 Stockholm, Sweden; 9 Division of Digestive and Liver Diseases, Department of Medicine, Irving Cancer Research Center, Columbia University Medical Center, New York, NY 10032, USA; 10 Division of Neurogeriatrics, Department of Neurobiology, Care Science and Society (NVS), Karolinska Institutet, 17177 Stockholm, Sweden

**Keywords:** Biological sciences, Molecular physiology, Molecular network

## Abstract

Enteroendocrine cells (EECs) differentiate and mature to form functionally distinct populations upon migration along the intestinal crypt-villus axis, but how niche signals affect this process is poorly understood. Here, we identify expression of Glial cell line-derived neurotrophic factor (GDNF) in the intestinal subepithelial myofibroblasts (SEMFs), while the GDNF receptor RET was expressed in a subset of EECs, suggesting GDNF-mediated regulation. Indeed, GDNF-RET signaling induced increased expression of EEC genes including *Tph1*, encoding for the rate-limiting enzyme for 5-hydroxytryptamine (5-HT, serotonin) biosynthesis, and increased the frequency of 5-HT+ enterochromaffin cells (ECs) in mouse organoid culture experiments and *in vivo*. Moreover, expression of the 5-HT receptor *Htr4* was enriched in Lgr5+ intestinal stem cells (ISCs) and 5-HT reduced the ISC clonogenicity. In summary, our results show that GDNF-RET signaling regulate EEC differentiation, and suggest 5-HT as a potential niche factor regulating Lgr5+ ISC activity, with potential implications in intestinal regeneration.

## Highlights

• GDNF is expressed in SEMFs and the GDNF receptor RET in enteroendocrine cells
• GDNF-RET signaling promotes enterochromaffin cell differentiation
• 5-HT reduces the organoid-forming capacity of Lgr5+ intestinal stem cells

## Graphical abstract

**Figure:**
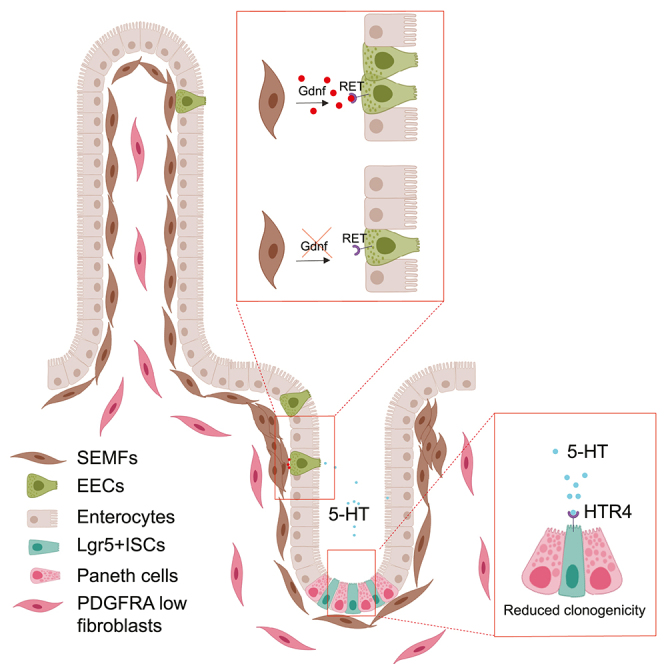

## Introduction

Intestinal epithelium undergoes constant and rapid turnover mandating a precise control of differentiation and stemness in strictly defined topological units. Accordingly, intestinal stem cells (ISCs) are situated in the epithelial crypt compartment adjacent to muscularis mucosa, while epithelial cell differentiation occurs during the migration of the cells toward the tips of the villi.^1^ The crypt base ISCs are marked by the expression of *Lgr5* and have the capacity to generate all differentiated cell types of the intestinal epithelium *in vitro* and *in vivo.*^2^^,^^3^ Of the differentiated intestinal epithelial cell (IEC) types, enteroendocrine cells (EECs) represent only about 1% and are scattered among the epithelium, but together form the body’s largest endocrine organ with major functions in the regulation of digestion and whole-body metabolism.^4^ Indeed, EEC-derived hormones and their receptors are under active investigation since they control e.g., food intake, gut motility, and mucosal immunity, thus representing potent drug targets for metabolic diseases, such as diabetes and obesity.^5^^,^^6^ At least 20 different peptides and other signaling molecules are derived from EECs.^7^ EECs include multiple subclasses with differential frequencies along the proximal-distal and crypt-villus axes.^8^^,^^9^ One subclass, enterochromaffin cells (EC), secrete 5-hydroxytryptamine (5-HT, serotonin) and are marked by the expression of tryptophan hydroxylase 1 (*Tph1*), the rate-limiting enzyme for 5-HT production. The young ECs in the crypt region are enriched for tachykinin (*Tac1*) expression, while secretin (*Sct*) -expressing ECs are more frequent in the villus, demonstrating hormone switching during maturation.^10^ L-cells express peptide YY (*Pyy*) and glucagon-like peptide (Glp1, encoded by *Gcg*) in the crypt area and maturate into cholecystokinin (*Cck*) and *Pyy*-expressing I-cells and further into neurotensin (*Nts*) and *Pyy*-expressing N-cells.^8^^,^^10^ The other EEC subclasses include K-cells expressing glucagon-like insulinotropic peptide (*Gip*), D-cells expressing somatostatin (*Sst*), and X-cells expressing ghrelin (*Ghrl*).

The maturation of intestinal epithelium is largely regulated by extrinsic cues from spatially distinct fibroblast populations secreting different growth factors along the crypt-villus axis.^11^ The most well-known differentiation signals, the bone morphogenic protein (BMP) ligands, are expressed in the crypt top fibroblasts,^12^ also known as *Pdgfra*^high^ fibroblasts,^13^ subepithelial myofibroblasts (SEMFs)^14^ or telocytes, and marked by the expression of *Foxl1*.^15^^,^^16^ Recent investigations have revealed that each EEC can produce several hormones and change their hormone expression patterns during the migration from the crypt to the villus,^10^ and the cell-intrinsic mechanisms affecting EEC maturation have been characterized.^17^^,^^18^^,^^19^ Of the extrinsic signals, the differentiation-promoting BMP ligands also promote EEC maturation,^10^ but it’s not clear if specific signaling cues from the tissue microenvironment affect only EECs. Neurotrophic factors (NTFs) are growth factors regulating the proliferation and differentiation of neuronal cells, and NTFs also have important non-neuronal roles including in the gastrointestinal tract.^20^ Since EECs share features with neurons,^21^ NTFs could represent candidate molecules shaping their maturation. Currently, the role of NTFs in regulating EECs remains uninvestigated.

Glial cell line-derived neurotrophic factor (GDNF) signals through rearranged during transcription (RET) tyrosine kinase receptor, and GDNF-RET signaling are essential for the development of the enteric nervous system and kidneys.^22^ In the adult intestine, RET is expressed in enteric neurons^23^ as well as in intestinal innate lymphoid cells where it is involved in gut defense.^24^ In addition, scattered expression of RET has been previously identified in intestinal epithelium where it was suggested to promote epithelial maturation through Wnt signaling.^25^ Interestingly, a recent study demonstrated functional importance for RET in the intestinal epithelium, but not in the enteric neurons, in regulation of the gut motility.^26^ The RET ligand GDNF is expressed in the developing mesenchyme where it regulates the migration and maturation of enteric neurons.^27^ Expression of GDNF has also been reported in intestinal glia^24^^,^^28^^,^^29^ and in the smooth muscle.^30^ However, antibody-based endogenous GDNF detection has triggered debate in the field over their specificity, and most antibodies used for research are not GDNF KO tissue verified. Thus, it remains to be established in which cells GDNF is expressed and what its biological role is in the adult gut.

With the capacity to monitor intestinal contents through nutrient receptors, EECs can relay luminal information to a wide variety of target cells through hormonal and paracrine interactions.^7^ Importantly, IECs express receptors for EEC-derived neurotransmitters and hormones, enabling EECs to regulate nutrient uptake.^31^^,^^32^^,^^33^^,^^34^^,^^35^^,^^36^^,^^37^^,^^38^ The Lgr5+ ISCs are also known to be regulated via EEC-derived signals,^39^^,^^40^ but the complete picture of the regulation is not clear. For example, it is unknown whether 5-HT, derived from ECs, directly regulates ISC behavior.

In this study, we address the role of GDNF-RET signaling in the maturation of EECs. Using bulk and single-cell RNA sequencing (scRNA-seq) analysis, mouse intestinal organoid culture, and *in vivo* models, we show that GDNF derived from *Pdgfra*^high^ SEMFs regulates the differentiation of EECs including ECs. Our results uncover a stromal-epithelial crosstalk mechanism governing intestinal epithelial maturation specifically in EECs and identify 5-HT as a potential niche factor affecting ISC activity.

## Results

### *Pdgfra*^high^ intestinal fibroblasts express GDNF

**Figure 1:**
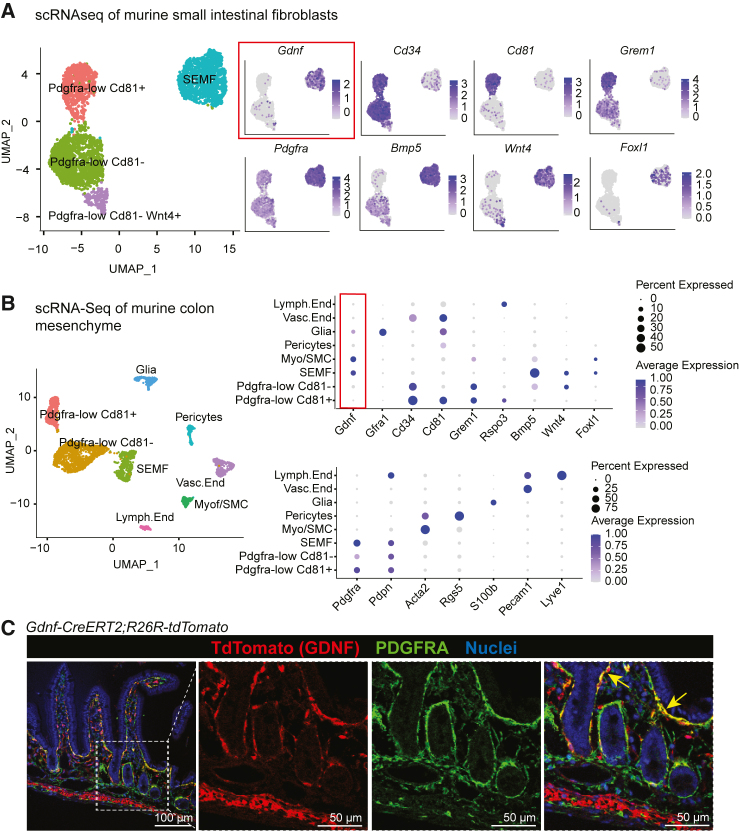
GDNF is expressed in the *Pdgfra*^high^ subepithelial myofibroblasts (A) Uniform manifold approximation and projection (UMAP) plots of Pdgfra+ intestinal fibroblast subsets^13^ (GEO: GSE130681). Expression patterns of indicated genes are shown. SEMF, subepithelial myofibroblasts. (B) UMAP (left) and dot plots (right) of a scRNA-seq dataset of colon mesenchymal cells^44^ (GEO: GSE142431). Markers used to identify fibroblast subpopulations (top dot plot) and mesenchyme cells subsets (bottom dot plot) are shown. Lymph.End, lymphatic endothelial cells; Vasc.End., vascular endothelial cells; Myof/SMC, myofibroblasts/smooth muscle cells; SEMF, subepithelial myofibroblast. (C) Lineage tracing of *Gdnf-Cre*^*ERT2*^*;Rosa26R-tdTomato* mice 10 days after tamoxifen delivery showing tdTomato (GDNF, red) and PDGFRA (green) expression in mouse jejunum. Yellow arrowheads mark the colocalization of tdTomato and PDGFRA. Scale bar: 100 µm and 50 μm for zoom-in images.

We hypothesized that NTFs from intestinal fibroblasts could impact EEC differentiation. To address this, we first investigated the expression patterns of a panel of NTFs in a bulk RNA-seq dataset of mouse intestinal epithelial and mesenchymal populations.^15^ Interestingly, we identified the expression of *Gdnf* in the *Foxl1*-expressing fibroblasts (Figure S1), suggesting a potential role in the regulation of the intestinal epithelium. Analysis of scRNA-seq data from mouse intestinal fibroblasts^13^ confirmed expression of *Gdnf* mRNA in the *Pdgfra*^high^ SEMF cluster also expressing *Foxl1*, *Bmp5*, and *Wnt4*,^15^^,^^41^ while it was absent in the stemness-supporting fibroblast populations expressing *Cd34*, *Grem1*, and *Cd81*^12^^,^^13^^,^^42^^,^^43^ (Figure 1A). To investigate *Gdnf* expression across mesenchymal cells, we also explored a dataset comprising non-fibroblast mesenchymal cells from mouse colon.^44^ Similarly to the intestine, *Gdnf* was expressed in the *Pdgfra*^high^, *Bmp+* SEMF cluster and was absent in the *Pdgfra*^low^ clusters. *Gdnf* expression was also noted in smooth muscle cells and glia, confirming previous reports^30^^,^^45^ (Figure 1B). To verify the expression site *in vivo*, we crossed *Gdnf-Cre*^*ERT2*^ knock-in mice^46^ to *R26R-LSL-tdTomato* reporter mice^47^ and induced recombination with tamoxifen, allowing tracing of GDNF-expressing cells. In accordance with the scRNA-seq data, TdTomato+ cells indicating *Gdnf* expression were noted in the inner smooth muscle layer, and in PDGFRA-positive fibroblasts adjacent to the epithelium, clustering predominantly at the crypt-villus junction (Figure 1C). These data demonstrate that GDNF is expressed in SEMFs, predominantly in the area separating the stem and progenitor cell-containing crypts from the differentiated cells in the villi.

### RET is expressed in EECs including late EEC progenitors

**Figure 2:**
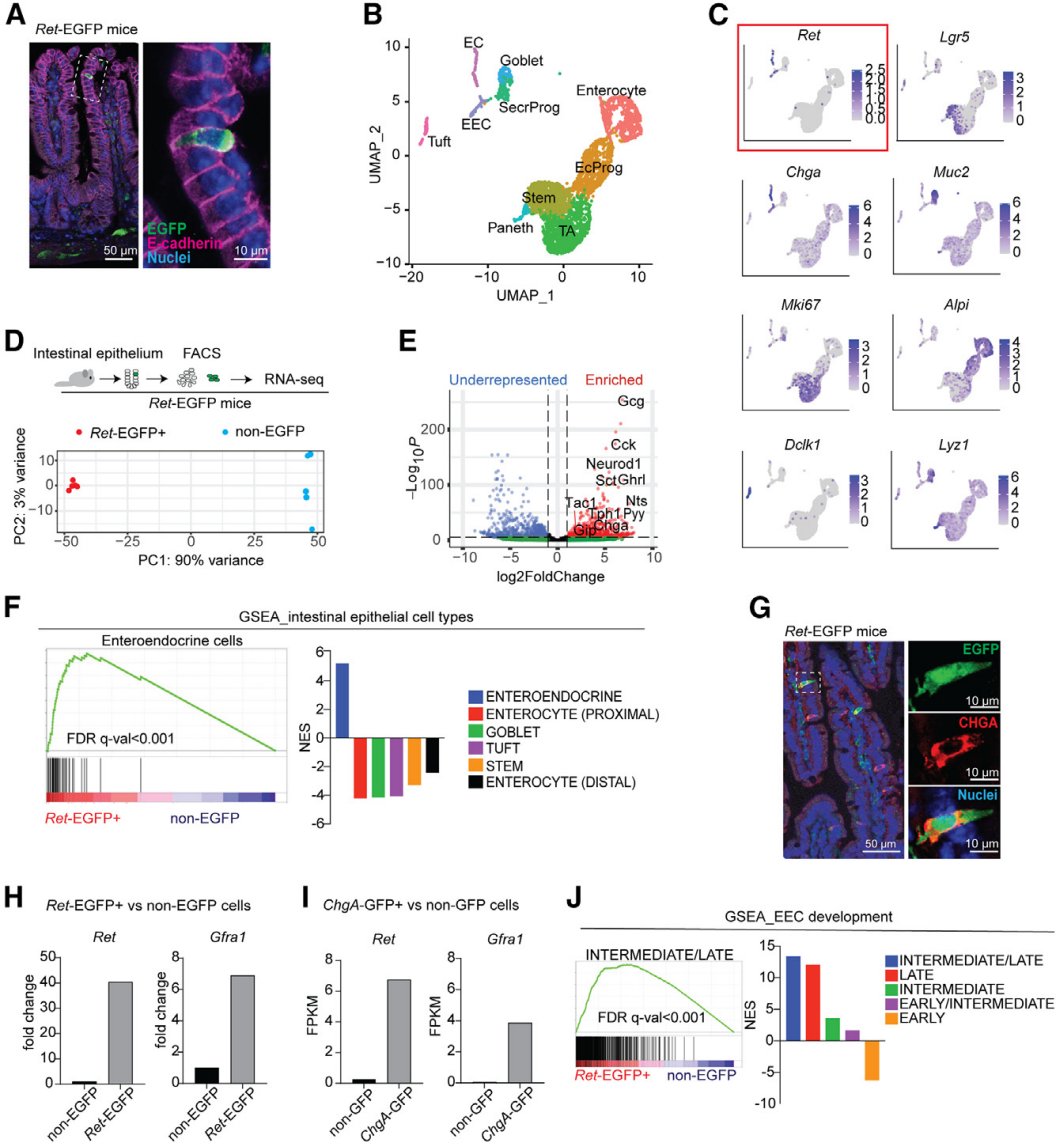
GDNF receptor RET is expressed in intestinal EECs (A) Immunofluorescence staining of EGFP (RET, green) and E-cadherin (magenta) in mouse jejunum. Scale bar: 50 μm. (B) UMAP plot of intestinal epithelial cell types^49^ (GEO: GSE92332). EC, enterochromaffin cells; EEC, enteroendocrine cells; SecrProg, secretory progenitors; EcProg, enterocyte progenitors; TA, transit-amplifying cells. (C) Expression patterns of indicated genes in the intestinal epithelial cell clusters. (D) Outline of the experiment (upper panel) and Principal component analysis (PCA) plot (bottom panel) of sorted *Ret*-EGFP+ cells (red dots) and non-EGFP cells (blue dots), *n* = 5 + 5 from 3 male mice. (E) Volcano plot of the differentially expressed genes in *Ret*-EGFP+ vs. non-EGFP cells. Blue and red dots represent significantly (*p* < 0.05) underrepresented (Log2FoldChange<−1) and enriched (Log2FoldChange>1) genes, respectively. EEC marker genes are highlighted. (F) Gene set enrichment analysis (GSEA) of the ranked gene list of *Ret*-EGFP+ cells compared to non-EGFP cells against intestinal epithelial cell type signatures.^49^ NES, normalized enrichment score. (G) Immunofluorescence staining of EGFP (RET, green) and Chromogranin-A (CHGA, red) in mouse jejunum. Scale bar: 50 μm for the left image and 10 µm for zoom-in images. (H) mRNA expression of *Ret* and *Gfra1* expression in sorted *Ret*-EGFP+ vs. non-EGFP IECs (this study). Fold change to non-EGFP cells is shown. (I) mRNA expression of *Ret* and *Gfra1* expression in sorted *Chga*-EGFP+ vs. non-EGFP IECs^51^ (GEO: GSE98794). FPKM, fragments per kilobase of transcript per million mapped reads. (J) GSEA analysis of sorted *Ret*-EGFP+ cell transcriptome against signatures identified in different phases of EEC maturation^55^ (GEO: GSE113561).

Since GDNF was expressed in SEMFs clustered in the crypt-villus junction in the intestinal epithelium, we next analyzed IECs for expression of the GDNF receptor RET. Analysis of intestinal tissues of *Ret*^*EGFP/+*^ knock-in mice^48^ revealed scattered E-cadherin-expressing EGFP+ cells, confirming RET expression in rare IECs (Figure 2A). In a mouse IEC scRNA-seq dataset,^49^ *Ret* expression was noted in Chromogranin A (*ChgA*) -expressing cells suggesting expression in EECs (Figures 2B and 2C). To get further insight into the identity of RET+ intestinal epithelial cells, we sorted EGFP+ and EGFP- cells from intestinal epithelium isolated from *Ret*^*EGFP/+*^ mice and performed bulk RNA sequencing (Figure 2D). The analysis of differentially expressed genes revealed strong enrichment of EEC-related genes in the EGFP+ cells (Figure 2E), and gene set enrichment analysis (GSEA) against IEC types confirmed specific enrichment of the EECs gene set (Figure 2F). Accordingly, EGFP-expressing cells also expressed CHGA in intestinal tissue sections (Figure 2G). The GDNF family receptor alpha 1 (GFRA1), the co-receptor required for GDNF-RET signaling, can be derived from RET-expressing cells (signaling in *cis*) or act as a soluble co-receptor and be produced by other adjacent cells (signaling in *trans*).^50^ *Gfra1* expression was enriched in RET-EGFP+ IECs (Figure 2H) as well as in CHGA+ IECs (Figure 2I),^51^ indicating an EEC-derived expression. In addition, mesenchymal *Gfra1* mRNA expression was noted in glial cells (Figure 1B), consistently with previous observations.^52^^,^^53^^,^^54^ Thus, the GFRA1 co-receptor is expressed in RET+ EECs, and soluble GFRA1 may be also available from adjacent mucosal glia. As a variety of different EEC-related transcripts were identified in RET+ cells (Figure 2E), indicating a possible progenitor identity, we next compared the transcriptional similarity of RET+ cells to different EEC progenitor stages. The transcriptional signatures along different stages of EEC development have been carefully documented using a dual fluorochrome reporter revealing the time after induction of the EEC-specific transcription factor *Neurog3*.^55^ RET+ cells showed the highest similarity with intermediate/late and late EEC progenitor signatures and were negatively enriched for early progenitor signature (Figure 2J), suggesting RET-signaling could regulate the fate decisions and maturation of EEC progenitors.

### GDNF induces EEC gene expression in intestinal organoids via RET

**Figure 3:**
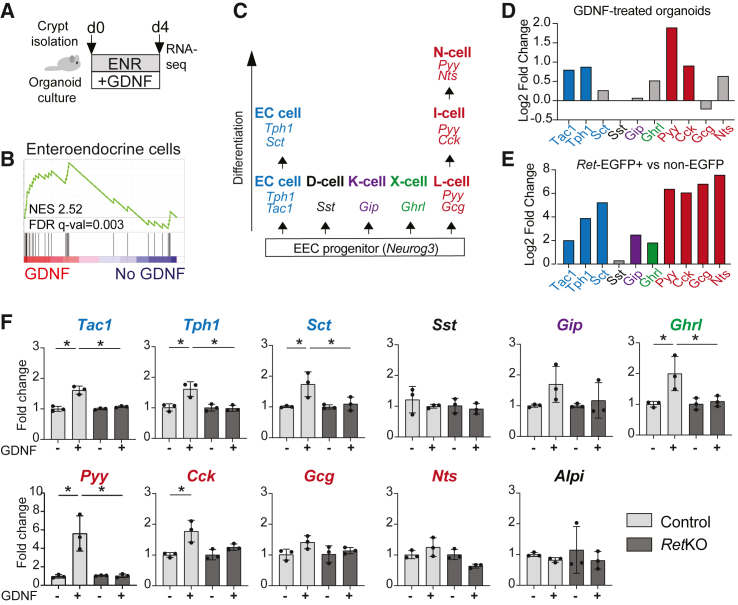
GDNF promotes EEC identity in intestinal organoids via RET (A) Outline of the RNA-seq experiment. (B) GSEA of the GDNF-induced transcriptome against the EEC signature gene set.^49^ (C) Schematic illustration of EEC lineage differentiation, including markers for each subtype. (D) Relative expression levels of indicated EEC markers in GDNF-treated organoids compared to control. Colored bars indicate adjusted *p* value <0.1; bar color refers to a representation of the EEC lineages as in C. (E) Relative expression levels of indicated EEC markers in sorted *Ret*-EGFP+ cells compared to non-EGFP intestinal epithelial cells, bar colors as in D indicate adjusted *p* value <0.1. (F) Relative expression level of indicated genes in control and *Ret* knock-out (KO) organoids with or without GDNF treatment. *N* = 3 repeats per treatment (∗*p* < 0.05, one-way ANOVA with Tukey’s post hoc test). Mean and standard deviation are shown.

To address the functional relevance of GDNF-RET signaling in the intestinal epithelium, we cultured mouse intestinal epithelial organoids in the standard ENR media^2^ or ENR supplemented with recombinant GDNF and GFRA1 and analyzed the GDNF-induced transcriptional changes using RNA-seq (Figure 3A). Interestingly, we observed a significant enrichment for EEC signature in GDNF-treated organoids (Figures 3A and 3B), including significant upregulation of *Tac1*, *Tph1*, *Pyy*, and *Cck* expression, suggesting that GDNF-RET signaling enhances the differentiation of ECs and L/I/N cell lineages (Figures 3C and 3D). Our results agreed with the ECs and L/I/N lineage cell marker genes showing the most significant enrichment in sorted *Ret*-EGFP+ cells (Figure 3E), and with a recent report indicating RET expression in ECs and L-cells.^26^ Furthermore, we investigated a scRNA-seq dataset of different mouse EEC subsets^55^ and verified high RET expression in one of the two EC populations, as well as in L/I/N-cells. Interestingly, in this dataset, RET was expressed also in X-cells (Figure S2). Importantly, GDNF promoted the EEC differentiation through the RET receptor, since GDNF treatment failed to increase the EEC marker expression in Ret knock-out (KO) organoids (Figures S3 and 3F). The increase in Wnt signaling activity, previously attributed to RET activity in the intestinal epithelium,^25^^,^^56^ was not seen in our assays (Figure S4) consistently with the known function of WNT signaling promoting stemness, not differentiation, of ISCs.

**Figure 4:**
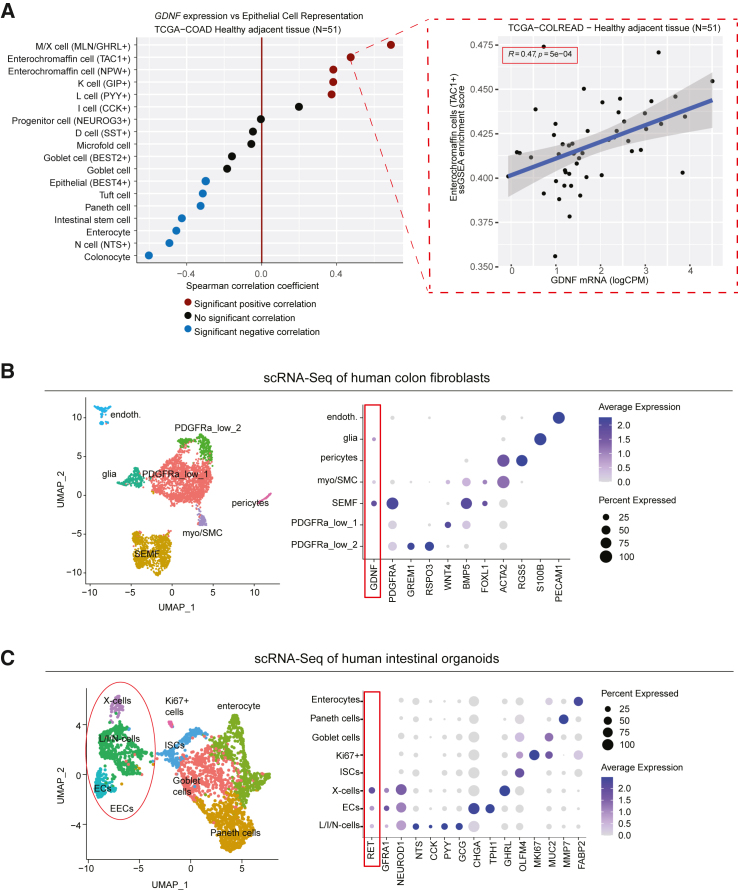
GDNF mRNA levels correlate with EEC signatures in the human colon (A) Left image: Dot plot depicting Spearman correlation coefficient between *GDNF* RNA expression and enrichment score for indicated intestinal epithelial cell types^58^ from healthy colon samples (*n* = 51) from the Cancer Genome Atlas.^57^ Red dots: significant positive correlation; blue dots: significant negative correlation (*p* < 0.01). Right image: close-up of enterochromaffin cell (TAC1+) enrichment score between *GDNF* RNA expression from 51 healthy colon samples. (B) UMAP and feature plots of *PDGFRA*, *FOXL1*, and *GDNF* in human colon mesenchyme scRNA-seq dataset^59^ (GEO: GSE114374). (C) UMAP and dot plots of indicated genes in human IECs^60^ (GEO: GSE146799).

To investigate the possible correlation of GDNF expression levels with EEC signatures in human tissue, we analyzed the healthy colon transcriptomes derived from the Cancer Genome Atlas (TCGA) cohort^57^ and compared the correlation of GDNF expression to human intestinal epithelium cell-type specific signatures.^58^ Indeed, all cell types with a significant positive correlation with GDNF levels (M/X-cells, ECs, K-cells, and L-cells), represented EECs (Figure 4A). Consistently, analysis of a human scRNA-seq dataset of colon mesenchyme^59^ revealed that similarly to mice (Figures 1A and 1B), *GDNF* was expressed in human *PDGFRA*^*high*^, *FOXL1*+, and *BMP5*+ cells representing SEMFs (Figure 4B). Analysis of the intestinal epithelial cells from human EEC Atlas^60^ revealed *RET* expression in human ECs, L/I/N-cells, and X-cells (Figure 4C), suggesting that the GDNF-RET pathway is involved in EEC differentiation also in humans. In summary, our results suggest that GDNF-RET signaling regulates the differentiation of some EEC subsets, including ECs and the L/I/N lineage, in both mice and humans.

### GDNF-RET signaling increases the density of 5-HT+ ECs

**Figure 5:**
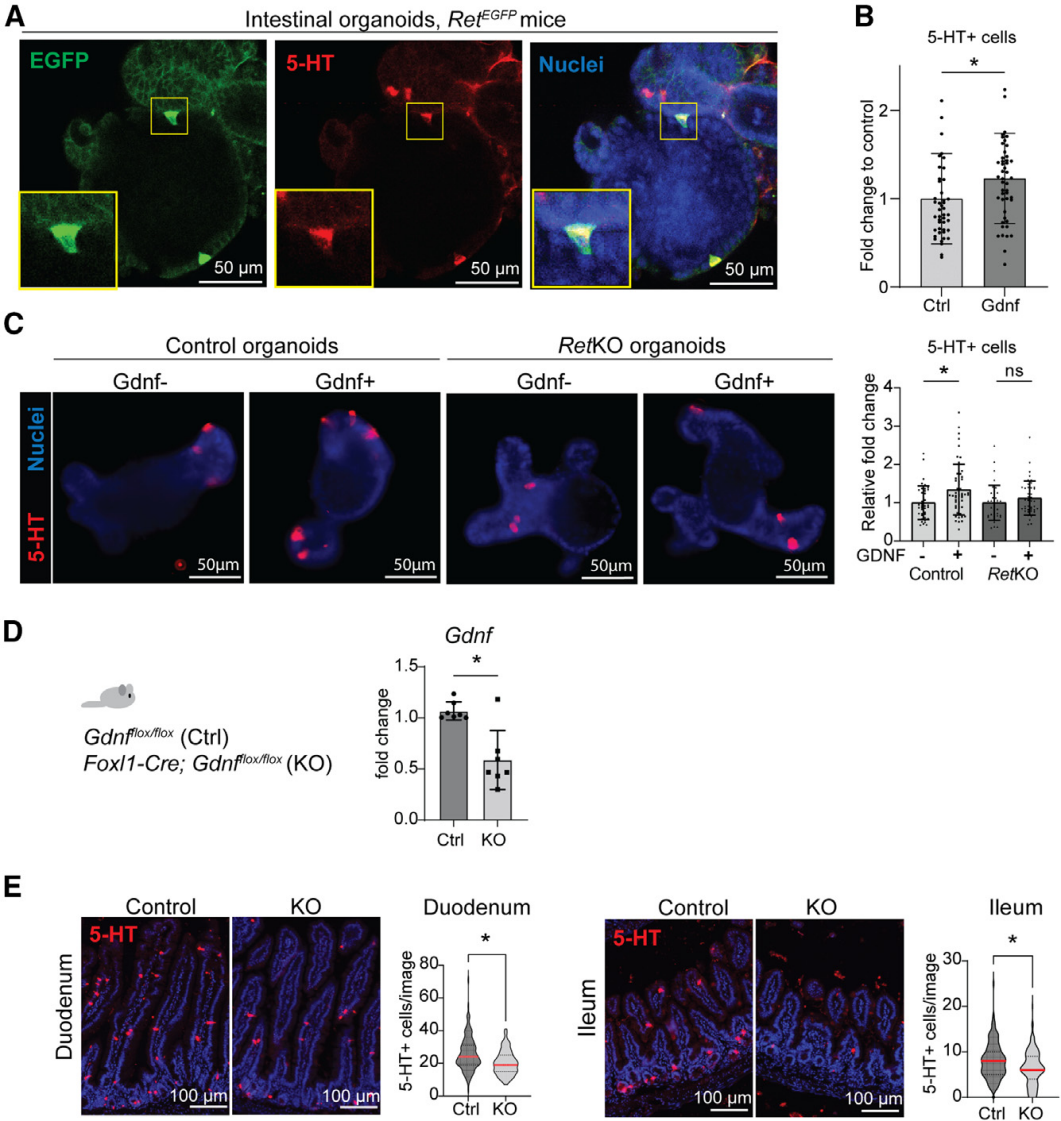
GDNF regulates the frequency of enterochromaffin cells *in vitro* and *in vivo* (A) Immunofluorescence staining of EGFP (RET) and 5-HT in *Ret*^EGFP^ mouse intestinal organoids. Zoom-in represents an example of co-localization. Scale bar: 50 μm. (B) Quantification of 5-HT+ cells in GDNF-treated organoids derived from WT male mice as compared to control organoids (Day 4). Relative fold change normalized to organoid size is shown, *n* = 46 control organoids and *n* = 48 GDNF-treated organoids were counted from 3 independent organoid cultures. Mean and standard deviation are shown. Asterisks indicate statistical significance (∗*p* < 0.05, two-tailed unpaired t test). (C) Representative images and quantification of 5HT + cells on control and *Ret*KO organoids with or without GDNF treatment. Control *n* = 40, Control+GDNF *n* = 53, *Ret*KO control *n* = 38, *Ret*KO+GDNF *n* = 43. (∗*p* < 0.05, one-way ANOVA with Tukey’s post hoc test). Mean and standard deviation are shown. Scale bar: 50 μm. (D) Gdnf mRNA expression in intestinal tissues of *Gdnf*^*flox/flox*^ (Ctrl, *n* = 7) and *Foxl1-Cre;Gdnf*^*flox/flo*x^ (KO, *n* = 7) mice. Mean and standard deviation are shown. Asterisks indicate statistical significance (∗*p* < 0.05, two-tailed unpaired t test). (E) Representative images and quantification of 5-HT positive cells in duodenum and ileum of control and KO mice. 5-HT positive cells in duodenum counted from at least 111 20× images derived from at least six individual mice. Duodenum: control, *n* = 148 images from *n* = 7 mice; KO, *n* = 111 images from *n* = 6 mice, *p* value=<0.0001. Ileum: 5-HT positive cells in ileum counted from at least 147 30× images control, *n* = 147 images; KO, *n* = 172 images from *n* = 7 mice, *p* value=<0.0001. Red line in the graph indicates the mean value and dashed lines indicate standard deviation. Asterisks indicate statistical significance (∗*p* < 0.05, two-tailed unpaired t test).

To further validate our transcriptomic analysis suggesting that GDNF-RET signaling regulates EC and L/I/N cell lineages, we stained 5-HT (a marker of EC) and PYY (a marker of L-cells) in the mouse intestinal organoids. Immunofluorescent staining of organoids derived from *Ret*^*EGFP*^ mice showed colocalization of EGFP with both 5-HT and PYY, confirming the expression of RET in EC and L-cell lineages (Figure 5A; Figure S5A). Interestingly, we observed that GDNF treatment significantly increased the number of 5-HT+ cells in a RET-dependent matter (Figures 5B and 5C) in organoid culture. Organoid culture experiments also showed a trend toward increased PYY+ cell number in response to GDNF treatment (Figure S5B), and a further analysis demonstrated a RET-dependent effect (Figure S5C). This indicates that the elevated expression of *Tph1* and *Pyy* in the intestinal organoids is associated with an increased number of 5-HT+ ECs and PYY+ L-cells. Next, we wanted to address whether GDNF could affect the number of 5-HT+ and PYY+ cells *in vivo.* For this we crossed *Gdnf*^*flox*^ mice with the *Foxl1-Cre* allele, specifically targeting GNDF expression in the intestinal subepithelial fibroblasts^61^^,^^62^^,^^63^ allowing enteric nervous system development and postnatal survival. Expression of *Gdnf* mRNA was reduced by approximately 50% in *Foxl1-Cre*;*Gdnf*^*flox/flox*^ intestines as compared to *Gdnf*^*flox/flox*^ littermates, suggesting that roughly half of the adult intestinal *Gdnf* expression is derived from *Foxl1-*expressing cells (Figure 5D). Consistently with the organoid culture experiments, the 5-HT+ ECs were significantly reduced in both the duodenum and ileum of *Foxl1-Cre*;*Gdnf*^*flox/flox*^ mice compared to the littermate controls (Figure 5E). PYY+ cells were slightly reduced in the duodenum while not altered in the ileum (Figure S5D). Notably, previous data indicates that ECs are regulated in a sex-dependent manner.^64^^,^^65^ Furthermore, gastrointestinal motility has been shown to be regulated by epithelial RET in male mice.^26^ In our cohort, more robust GDNF-mediated regulation of EECs was observed in females (Figures S6A and S6B), suggesting possible sex-dependent effects in the EEC differentiation that should be validated in larger cohorts. There were no differences in the bodyweight, total epithelial area, intestine length, villus height, crypt depth or the overall structure of the enteric nervous system between the mouse genotypes (Figure S7). In summary, these results indicate that GDNF-RET signaling regulates the density of ECs in the intestine.

Interestingly, increased GDNF levels and 5-HT+ cell frequency have been previously connected to intestinal inflammation.^56^^,^^66^^,^^67^ We noted that the inflammation-inducing agent lipopolysaccharide (LPS) induced increased *Gdnf* expression in cultured primary intestinal fibroblasts (Figure S8). This suggests, that inflammation-induced increase of GDNF in SEMFs may be associated with the increased levels of 5-HT observed in inflammatory conditions.^56^^,^^67^^,^^68^^,^^69^

### 5-HT affects the clonogenicity of Lgr5+ ISCs through HTR4

**Figure 6:**
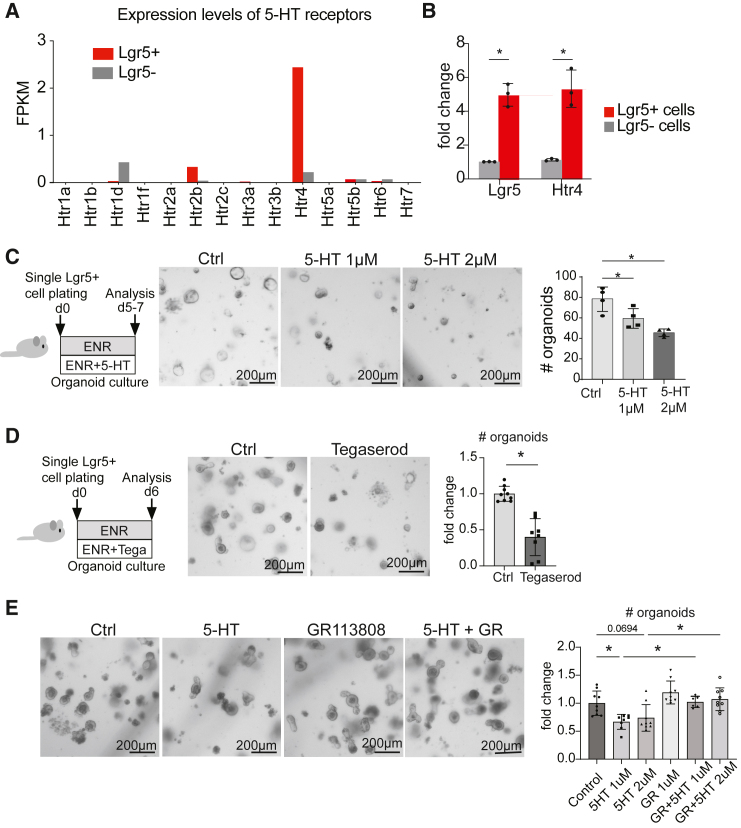
5-HT reduces Lgr5+ ISC clonogenicity via HTR4 (A) Expression levels of all 5-HT receptors in RNA-seq data from Lgr5+ and Lgr5- IECs^75^ (GEO: GSE99457). FPKM; Fragments Per Kilobase of transcript per Million mapped reads. (B) Relative expression of *Lgr5* and *Htr4* mRNAs in sorted Lgr5-EGFP+ and Lgr5-EGFP- IECs measured using qPCR. Each dot represents an individual mouse (*n* = 3), (∗*p* < 0.05, two-tailed unpaired t test). Mean and standard deviation are shown. (C) Outline of the experiment (left), representative images (middle), and quantification of control and 5-HT-treated organoids (right). A representative of *n* = 4 experiments is shown; at least 3 technical replicates were analyzed per experiment. Asterisks indicate statistical significance (∗*p* < 0.05, one-way ANOVA with Tukey’s post hoc test). Mean and standard deviation are shown. Scale bars: 200 μm. (D) Outline of the experiment (left), representative images (middle), and quantification of Tegaserod-treated (1 μM) organoid formation compared to control organoids (∗*p* < 0.05, two-tailed unpaired t-test), *n* = 3 experiments with 3 replicate wells. Mean and standard deviation are shown. Scale bars: 200 μm. (E) Representative images of single Lgr5+ cells grown for 6 days in indicated conditions. GR, GR113808 (1 μM), 5-HT (2 μM). Quantification of the organoid formation was done using the organoid classifier tool Tellu.^80^*n* = 3 experiments with 3 replicate wells, except GR+5HT 1μM condition with *n* = 2 independent experiments. Asterisks indicate statistical significance (∗*p* < 0.05, one-way ANOVA with Tukey’s post hoc test). Mean and standard deviation are shown. Scale bars: 200 μm.

EECs are known to exert their functions through both hormonal and paracrine effects, and ablation of EECs affects the proliferation and function of ISCs.^70^ Few EEC-derived factors such as Glp-2 and Nts in mammals^39^^,^^40^ as well as tachykinin and neuropeptide Bursicon in flies,^71^^,^^72^ are known to regulate ISC activity. Interestingly, neuronal 5-HT has been connected to increased ISC activity via indirect mechanisms,^73^^,^^74^ but the direct regulation of Lgr5+ ISCs by 5-HT has not been reported. Here, we set out to investigate if 5-HT from GDNF-RET-regulated ECs impacts intestinal epithelial dynamics through stem cell regulation. First, we analyzed the expression patterns of all fourteen 5-HT receptors in previously published datasets of sorted Lgr5+ ISCs^75^^,^^76^^,^^77^ and found an ISC-enriched expression of the 5-HT receptor 4 (*Htr4*) (Figure 6A; Figures S9A and S9B). We confirmed the enrichment of *Htr4* mRNA expression in sorted Lgr5+ ISCs by qPCR (Figure 6B), consistent with previous reports showing colocalization of *Htr4* and *Lgr5* in the intestinal and gastric mucosa.^78^^,^^79^ Different serotonergic receptors are expressed in all tissues across cell types, complicating functional experiments addressing the direct 5-HT-induced effects in a certain cell type using *in vivo* models. Thus, we addressed the role of 5-HT in regulating Lgr5+ ISC activity by measuring the ability of isolated single Lgr5+ ISCs to form intestinal organoids. Intriguingly, the organoid-forming capacity of Lgr5+ ISCs was significantly reduced upon 5-HT treatment, without affecting the size of organoids (Figures 6C and S9C), and a similar result was observed when the ISCs were treated with the HTR4 agonist Tegaserod (Figure 6D). In addition, the potential of 5-HT to reduce the Lgr5+ ISC clonogenicity was reversed in the experiments where 5-HT was added in combination with the HTR4 antagonist GR113808 (Figure 6E). These results suggest that 5-HT regulates the clonogenicity of the Lgr5+ ISCs through HTR4.

## Discussion

EEC differentiation has been shown to be promoted by activation of BMP signaling, inhibition of RhoA signaling, or by combined inhibition of EGFR/WNT/Notch receptors.^10^^,^^19^^,^^81^ However, subepithelial niche-derived growth factors with specific activity toward EECs have not been described. Furthermore, EECs are a heterogeneous class of cells with at least 5 distinct lineages,^8^ and it is not known if specific signals from microenvironment impact EEC differentiation. Since EECs share features with neurons,^21^ we were interested to study the role of NTFs in regulating EEC differentiation. We show that GDNF-RET signaling regulates the differentiation of EECs, especially toward the 5-HT-producing ECs. Thus, we describe a postnatal function for intestinal GDNF, which is mostly known for its crucial role in promoting the migration and maturation of enteric neurons during development.^27^ Interestingly, GDNF was previously shown to promote development of pancreatic beta-cells,^82^ suggesting a wider role in endocrine cell regulation, consistently with expression of RET in different endocrine cell populations.^83^^,^^84^ The transcriptome of RET+ IECs showed overlap with late EEC progenitors and subsets of mature EECs, suggesting that GDNF-RET signaling is not important in the early EEC differentiation unlike in the development of enteric nerves^85^^,^^86^ but rather play a role in the fine-tuning of EEC populations. EEC differentiation has also been shown to be regulated through microbiome-derived short-chain fatty acids,^87^^,^^88^ suggesting microbiome as an important contributor to EEC differentiation processes, especially in the microbe-rich colon. The possible complementary effects of microbial products and GDNF signaling would be an interesting avenue for future investigation.

Our organoid culture experiments showed that GDNF treatment robustly increased the expression of L/I/N lineage peptide transcripts, especially *Pyy* (Figure 3D). However, the PYY+ cell number was only marginally affected by GDNF treatment in the organoids and the SEMF-specific GDNF deletion *in vivo* (Figures S5B–S5D). This implies that GDNF-RET signaling may impact the PYY levels within the pre-existing L-cells, rather than regulating the cell number. The signaling could also impact the development of neuropods, the axon-like processes that contain most of the peptide-containing vesicles in PYY+ cells and have been reported to be regulated by other NTFs.^89^ Interestingly, a recent study addressed the etiology of Hirschprung’s disease by deleting RET in intestinal neurons and IECs. The study revealed that RET co-localized with 5-HT and PYY in the intestinal epithelium, consistently with our results.^26^ Moreover, epithelium-specific loss of RET was associated with enhanced amounts of postprandial circulating PYY and increased intestinal transit time in male mice, suggesting a causal role for epithelial RET in Hirschprung’s disease.^26^ Our observation that GDNF-RET signaling increases *Pyy* transcription, coupled with the previous finding that the absence of RET results in increased circulating PYY, suggests a developmental role for RET in EEC development and a complex regulatory mechanism of PYY production and release involving the GDNF-RET pathway. Interestingly, our results suggest a more robust role for GDNF-RET signaling to EEC differentiation in females as compared to males, and it would be interesting to investigate the sex-specific effects further using larger mouse cohorts.

GDNF-RET signaling induced the expression of *Tph1* and increased the 5-HT+ EC density in the intestinal epithelium, indicating an important function in regulating EC development. Interestingly, and consistent with our findings, intraperitoneal injection of recombinant GDNF was previously shown to increase the number of ECs in murine colon.^56^ 5-HT is released from ECs after mechanical or chemical stimuli and signals to enteric neurons to activate peristaltic reflexes.^90^ In addition, 5-HT can be distributed systemically via circulation, where it is stored mostly in platelets, or act in a paracrine fashion affecting adjacent cells. 5-HT regulates target cells via the 14 different 5-HT receptors expressed across different cell types to e.g., coordinate inflammatory processes.^91^ Surprisingly, Htr4 was the most highly expressed 5-HT receptor and significantly enriched in the Lgr5+ IECs, indicating possible direct regulation of IECs by 5-HT. Previously, the effects of neuronal and epithelial 5-HT on intestinal epithelium growth have been studied using KO mice for *Tph1* (essential for 5-HT biosynthesis in ECs) or *Tph2* (essential for 5-HT biosynthesis in neurons). Interestingly, *Tph2* KO mice displayed reduced proliferation in the intestinal epithelium, suggesting a stimulatory function for neuronal 5-HT.^73^^,^^74^^,^^92^ However, the neuronal 5-HT did not act directly on epithelial cells, but drove proliferation via activation of cholinergic neurons^73^ and enteric macrophages.^74^ Recently, colorectal cancer stem cells were suggested to be directly regulated by neuronal 5-HT via HTR1B/D/F.^93^ Our finding that 5-HT reduces the clonogenicity of wild-type Lgr5+ISCs via HTR4 implies that 5-HT could also play a direct role in the homeostatic regulation of intestinal epithelium. Using organoids as tools to address intestinal epithelial growth allows assessment of IEC without potential confounding effects from other cell types expressing 5-HT receptors. However, this system does not consider the cellular origin of the 5-HT. Interestingly, deletion of *Tph1*, resulting in a lack of EEC-derived 5-HT, was previously shown to be associated with increased crypt depth and villus height, suggesting potential negative regulation of ISCs by EEC-derived 5-HT.^73^ This would be consistent with the regulation of ISCs being a result of epithelial 5-HT. In the future, it would be interesting to combine *Tph1* and *Tph2* KO alleles with intestinal epithelium-specific *Htr4* KO to investigate the *in vivo* relevance of IEC-specific 5-HT in IECs via HTR4. Also, further investigation is needed to determine if inflammation-induced upregulation of the GDNF levels in SEMFs is sufficient to control physiological levels of 5-HT and ISC clonogenicity.

5-HT signaling induces gut inflammation by elevating levels of proinflammatory factors^94^ and recruiting innate immune cells, such as neutrophil intravasation.^95^ Consistently, inhibition of TPH1 in mice ameliorated gut inflammation.^96^ We noted an increased GDNF expression in LPS-treated primary intestinal fibroblasts, and similar effects were previously established for enteric glia^24^ and macrophages.^97^ This suggests that inflammation-induced increase of GDNF expression in the intestinal mucosa could be linked to alterations in EEC maturation. For example, increased 5-HT-mediated suppression of Lgr5+ ISC activity could be physiologically relevant in early inflammatory steps of regeneration, where ISC activity is temporarily suppressed.^98^

In summary, our results identify GDNF-RET-axis as a stroma-epithelium crosstalk pathway regulating intestinal EECs, complementing the existing body of literature describing fibroblast-derived niche factors impacting IEC stemness and maturation.^12^^,^^13^^,^^15^^,^^42^^,^^43^^,^^63^ We also report that 5-HT reduces the clonogenicity of Lgr5+ ISCs, opening a potential new avenue for investigating ISC biology.

### Limitations of the study

Limitations of this study include the reliance on mouse models and organoids to demonstrate the impact of GDNF-RET signaling on EC differentiation. The extrapolation of these findings to human tissues is based on correlative gene expression patterns and has not been experimentally validated in human intestinal organoids. Additionally, the biological significance of the observed reduction in Lgr5+ISC clonogenicity by 5-HT is not fully understood. While it is possible that this mechanism may contribute to the temporary quiescence of Lgr5+ISC during regeneration, its *in vivo* relevance remains uncertain. Furthermore, the relationship between GDNF levels and the extent of 5-HT release from ECs has yet to be elucidated. The primary cell experiments were performed using cells derived from both male and female mice and were not analyzed separately, therefore we cannot address the influence of sex in primary cell cultures.

## Resource availability

### Lead contact

Requests for further information and resources should be directed to and will be fulfilled by the lead contact, Saara Ollila (saara.ollila@helsinki.fi).

### Material availability

The study did not generate new unique reagents.

### Data and code availability

• Bulk-RNA-seq data have been deposited at Gene Expression Omnibus as GSE259326 and are publicly available as of the date of publication.
• This paper does not report any original code.
• Any additional information required to reanalyze the data reported in this paper is available from the lead contact upon request.

## Acknowledgments

The library preparation and 3′ RNA sequencing were performed by Biomedicum Functional Genomics Unit, confocal microscopy was performed at the Biomedicum Imaging Unit, flow cytometry at the Biomedicum Flow Cytometry Unit, and scanned images were generated using the 3DHISTECH Pannoramic 250 FLASH II digital slide scanner at the Genome Biology Unit, all units supported by the Helsinki Institute of Life Science (HiLIFE) and 10.13039/501100013840Biocenter Finland at the University of Helsinki. Part of this work was carried out with the support of HiLIFE Laboratory Animal Centre Core Facility, 10.13039/100007797University of Helsinki, Finland. Graphical abstract was created with BioRender.com. We thank Dr. Eva Domènech-Moreno for the insightful discussions and help with bioinformatics. We thank MSc Reeta Huhtala, MSc Riikka Saikkonen, MSc Myriam Sevigny, MSc Melissa Montrose, and Bryana Belin for assistance in the project. We also thank Dr. Lauriina Porokuokka for collecting tissues from *Gdnf-Cre*^*ERT2*^;*R26R-LSL-tdTomato* mice for the project. All authors had access to the study data and had reviewed and approved the final manuscript. This work was funded by the 10.13039/501100003125Finnish Cultural Foundation (grant number 00240777), 10.13039/501100002341Research Council of Finland (grant number 317239), Instumentarium Science Foundation, and from Sigrid Jusélius Fellowship.

## Author contributions

Conceptualization: T.T.L. and S.O.; methodology: T.T.L., S.O., E.W.V., L.W., and P.P.; investigation: T.T.L., E.W.V., L.W., P.P., H.T.V., N.P., and S.O.; resources: T.P.M., P.K., T.C.W., J.-O.A., and S.O.; writing—original draft: T.T.L. and S.O.; writing—review and editing: E.W.V., L.W., P.P., N.P., T.P.M., T.C.W., and J.-O.A.; visualization: T.T.L., E.V., and S.O.; supervision: S.O.; project administration: S.O.; funding acquisition: T.T.L. and S.O.

## Declaration of interests

The authors declare no competing interests.

## STAR★Methods

### Key resources table

**Table:** 

REAGENT or RESOURCE	SOURCE	IDENTIFIER
**Antibodies**		

GFP	Abcam	Cat# ab290; RRID:AB_2313768
Rabbit Anti-Peptide YY/PYY Polyclonal Antibody, Unconjugated	Bioss	Cat# bs-2265R; RRID:AB_10857191
Goat Anti-Serotonin Polyclonal Antibody, Unconjugated	Abcam	Cat# ab66047; RRID:AB_1142794
Mouse PDGF R alpha Antibody	R&D Systems	Cat# AF1062; RRID:AB_2236897
RFP Antibody Pre-adsorbed	Rockland	Cat# 600-401-379; RRID:AB_2209751
Chromogranin A antibody	Abcam	Cat# ab45179; RRID:AB_726879
Monoclonal Anti-Uvomorulin/E-Cadherin antibody produced in rat	Sigma-Aldrich	Cat# U3254; RRID:AB_477600
Donkey Anti-Rabbit IgG (H + L) Antibody, Alexa Fluor 488 Conjugated	Molecular Probes	Cat# A-21206; RRID:AB_2535792
Donkey Anti-Goat IgG (H + L) Antibody, Alexa Fluor 594 Conjugated	Molecular Probes	Cat# A-11058; RRID:AB_142540
Donkey Anti-Rabbit IgG H&L (Alexa Fluor® 488) preadsorbed	Abcam	Cat# ab150065; RRID:AB_2860569
Anti-beta III Tubulin antibody - Neuronal Marker	Abcam	Cat# ab18207; RRID:AB_444319

**Chemicals, peptides, and recombinant proteins**		

recombinant human GDNF	Icosagen	Cat# P-103-100
recombinant human GFRA1	Icosagen	Cat# P-120-100
EGF	Gibco	Cat# PMG8041
NOGGIN	Peprotech	Cat# 250-38
R-spondin 1	R&D systems	Cat# 3474-RS
Y-27632	Sigma-Aldrich	Cat# Y0503
N-acetyl-L-cysteine	Sigma-Aldrich	Cat# A9165
Serotonin hydrochloride	Tocris	Cat# 3547
Tegaserod maleate	Sigma-Aldrich	Cat# SML1504
GR113808	Sigma-Aldrich	Cat# G5918

**Critical commercial assays**		

Nucleospin RNA Plus isolation kit	Macherey-Nagel	Cat# 740984.250
Mycoplasmacheck Eurofins	Eurofins	49167719

**Deposited data**		

Data of bulk RNA-Seq	This Paper	GSE259326
scRNA-seq data of murine small intestine fibroblasts	McCarthy et al.^13^	GSE130681
scRNA-seq data of murine colonic mesenchymal cells	Roulis et al.^44^	GSE142431
scRNA-seq data of murine small intestinal epithelial cells	Haber et al.^49^	GSE92332
scRNA-seq data of murine EECs	Gehart et al.^55^	GSE113561
scRNA-seq data of human colon mesenchyme	Kinchen et al.^59^	GSE114374
scRNA-seq data of human intestinal organoids	Beumer et al.^60^	GSE146799

**Experimental models: Cell lines**		

HEK293FT	ATCC	RRID:CVCL_6911

**Experimental models: Organisms/strains**		

Mouse:C57BL/6J	The Jackson Laboratory	RRID:IMSR_JAX:000664
Mouse: B6.129(Cg)-Rettm13.1Jmi/SjnJ	The Jackson Laboratory	RRID:IMSR_JAX:029847
Mouse: B6.129P2-Lgr5tm1(cre/ERT2)Cle/J	The Jackson Laboratory	RRID:IMSR_JAX:008875
Mouse: B6;SJL-Tg(Foxl1-cre)1Khk/J	The Jackson Laboratory	RRID:IMSR_JAX:017738
Mouse: B6.129S1(Cg)-Gdnftm1.1Neas/J	The Jackson Laboratory	RRID:IMSR_JAX:014097
Mouse: B6;129S6-Gdnftm1(cre/ERT2)Cos/J	The Jackson Laboratory	RRID:IMSR_JAX:024948
Mouse: B6.Cg-Gt(ROSA)26Sortm14(CAG-tdTomato)Hze/J	The Jackson Laboratory	RRID:IMSR_JAX:007914

**Oligonucleotides**		

Guide RNA against Ret 5′CAATCTTGCGGCTGTCACGG3′	Eurofins Genomics	N/A
See Table S1 for all qPCR primers	Eurofins Genomics	N/A

**Recombinant DNA**		

LentiCRISPRv2 plasmid	Addgene	RRID:Addgene_52961

**Software and algorithms**		

ImageJ	Schindelin et al.^99^	https://imagej.net/software/fiji/
R project	The R project for Statistical Computing	https://www.r-project.org/
Seurat v4 pipeline	Hao et al.^100^	https://satijalab.org/seurat/articles/get_started.html
GSEA v4 software	Subramanian et al.^101^	https://www.gsea-msigdb.org/gsea/index.jsp

### Experimental model and study participant details

#### Mice

Mouse alleles and origins: *Ret-EGFP* (Strain No: 029847),^48^ *Lgr5-EGFP-IRES-Cre*^*ERT2*^ (Strain No: 008875),^3^ *Foxl1-Cre* (Strain No: 017738)^61^ *Gdnf*^*flox*^ (Strain No: 014097),^62^ *Gdnf-Cre*^*ERT2*^ (Strain No: 024948),^46^ and *R26R-LSL-tdTomato* (Strain No: 007914)^47^ were purchased from Jackson laboratories. Mice were maintained in C57BL/6J (Stain No: 000664) background except for *Foxl1-Cre* and *Gdnf-Cre*^*ERT2*^ whose background was mixed as detailed in the mouse strain overview form the Jackson Laboratory.

Animals were housed and taken care of at the University of Helsinki Laboratory Animal Center according to national and international legislation and guidelines (licenses: ESAVI/8804/2020; ESAVI/3816/2023; KEK-23-006) approved by the Finnish Regional State Administrative Agency. Mice had free access to food and water. Mice were housed in individually ventilated cages containing 2–4 mice per cage, except in rare cases where single housing was needed. Mice were housed in a specific pathogen-free facility. Sex of the mice used for each experiment is detailed in figure legends and/or in the more detailed methods below. All experiments were performed on adult mice (2–10 months of age). Age-matched littermate controls were used in all the experiments.

For counting PYY+ and 5-HT+ cells from the intestinal tissue, a cohort of *Foxl1-Cre;Gdnf*^*flox/flox*^ mice, and littermate controls (*Gdnf*^*flox/flox*^) was generated.

For tracing GDNF-expressing cells, *Gdnf-Cre*^*ERT2*^*;R26R-LSL-tdTomato* mice were used. Recombination in *Gdnf-Cre*^*ERT2*^ mice was induced using intraperitoneal injection of 100 mg/kg Tamoxifen (Sigma-Aldrich, T2859) in corn oil (Sigma-Aldrich, C8267) for 5 consecutive days. Mice were euthanized 10 days after the last tamoxifen injection.

#### Human cell line

HEK293FT cell line from ATCC (CRL-1573) is human kidney cells derived from female embryo. Cells were seeded in DMEM, 10% FBS and 1% L-glutamate at 37°C in 5% CO_2_. The cells were tested for mycoplasma contamination (Mycoplasmacheck Eurofins, 49167719) but not authenticated for this study.

#### Primary intestinal fibroblast culture

Primary intestinal fibroblasts were isolated from wild-type C57BL/6J mice as detailed below in “primary intestinal fibroblasts culture” -section and cultured in fibroblast growth medium (DMEM containing 20% fetal bovine serum, 1% penicillin-streptomycin, and 1% L-glutamine) at 37°C in 5% CO_2_. Lipopolysaccharide (LPS) (L4516, Sigma-Aldrich) was used at 1000 ng/mL. The media was changed every 2–3 days.

#### Primary intestinal organoid culture

Intestinal crypts were isolated from wild-type C57BL/6J mice as detailed below in the “isolation of intestinal crypts” -section. Both male and female mice were used. The age of the mice from which primary cells were collected ranged from 2 to 10 months. Organoids were cultured in organoid culture media composed of Advanced Dulbecco’s modified Eagle medium (DMEM):F12 (12634010, Gibco) supplemented with 1M HEPES pH 7.4 (H0887, Sigma-Aldrich), 1× GlutaMAX (35050061, Gibco), 1× penicillin-streptomycin (P4333, Sigma-Aldrich), 1× *N*-2 supplement (17502048, Gibco) and 1× B-27 supplement (17504044, Gibco). The crypts in the basal medium were mixed in a 1:3 ratio with Matrigel (356231, Corning) before plating 20μL domes to 48-well plates. The domes were overlaid with ENR medium, composed of organoid growth media supplemented with EGF (Gibco, PMG8041; 50 ng/mL), NOGGIN (Peprotech, 250-38; 100 ng/mL), R-Spondin 1 (R&D Systems 3474-RS; 250 ng/mL) and N-acetyl-L-cysteine (A9165, Sigma-Aldrich; 1μM). Y-27632 (Y0503, Sigma-Aldrich; 10μM) was added in the first 2-3 days to initiate growth. When indicated, GDNF (P-103-100, Icosagen) and GFRA1 (P-120-100, Icosagen) at 100 ng/mL; except for bulk RNA-seq experiment at 500 ng/mL, were added to the organoid growth media. The media was changed every 2–3 days. Organoids were grown at 37°C in 5% CO_2_.

#### Lgr5+ single cell culture

Lgr5+ single cells were isolated from *Lgr5-EGFP-IRES-Cre*^*ERT2*^ mice (Strain No: 008875)^3^ as detailed below in “isolation and culture of single Lgr5-EGFP cells” -section. Single cells were mixed with Matrigel (356231, Corning) in a 1:3 ratio and cultured with the same ENR media as organoids, except that the media included 1 μg/mL RSPO1 (3474-RS, R&D Systems) and 2.5 μM CHIR99021 (SML1046, Sigma) for the first 2–3 days. Serotonin hydrochloride (3547, Tocris), Tegaserod maleate (SML1504, Sigma), and GR113808 (G5918, Sigma) were used for the serotonin experiments. Serotonin was diluted in water, Tegaserod maleate, and GR113808 in DMSO and used as described in the figure legends. The media was changed every 2–3 days. Single cells were grown at 37°C in 5% CO_2_.

### Method details

#### Isolation of intestinal crypts

Intestinal organoids were cultured with a modified version of a previously published protocol.^2^ The small intestine was removed, flushed with ice-cold PBS, and opened longitudinally. After removal of extensive mucus, the intestine was cut into 2-3mm fragments that were placed in a 50mL tube with PBS on ice and shaken gently. The tissue fragments were let to settle to the bottom and the supernatant was replaced by 20mL of 10mM EDTA in PBS. The tube was placed on ice horizontally and shaken gently for 1h 45min and the buffer was changed three times during the first 45min. To detach the crypts, the tube was shaken vigorously for 15s, and the detached crypts were filtered through a 70μm cell strainer and collected by centrifugation (200*g* for 2min). The pellet was washed with ice-cold PBS and collected as above. The crypts were resuspended in ENR media and mixed with Matrigel before plating (see above).

#### Isolation and culture of single Lgr5-EGFP cells

Intestinal crypts from *Lgr5-EGFP-IRES-Cre*^*ERT2*^ mice were collected as detailed above. The crypts were dissociated to single cells using 1 mg/mL DNase 1 (10104159001, Roche) in TrypLE Express (12605-010, Thermo Fisher Scientific) for 3 min in +37°C water bath followed by trituration through a 1 mL pipet on ice for 12 times. The dissociated cells were suspended into 5 mL of organoid growth media (see above) containing 10 μM Y-27632 and 1 μM n-acetylcysteine, filtered through a 40 μm strainer and spun down at 200 × g for 5 min. The pellet was resuspended in the same media as before with the addition of 1% bovine serum albumin (P6154-100GR, Biotop) and filtered again through a cell-strainer cap into a 5 mL tube (352235, Corning). 1 mM of EDTA was added to prevent cells from clumping. EGFP+ cells were sorted with a Sony SH800z cell sorter. The gate was set using crypt cells isolated from control (non-EGFP expressing) mice. After the sorting, cells were spun down 200 × g for 5 min and 400 × g for 1 min and plated to Matrigel (354234, Corning) domes (8 ul each) containing 5000 EGFP+ cells each as described above. Quantification for Figures 6E and S9C was done from 4× images using an unbiased organoid counting tool.^80^

#### Isolation and sequencing of single *Ret-EGFP*+ cells

Single cells were isolated from crypts of *Ret-EGFP* mice as described above and sorted with the Sony SH800z cell sorter using an age-matched littermate control to set the gate for EGFP+ cells. The RNA sequencing method from RNAlater preserved cells was designed based on the Drop-seq protocol described in.^102^ Briefly, a total of 1000 cells per sample (1–2 samples/mouse, total *n* = 5 + 5 samples from *n* = 3 male mice) were sorted directly on a 96-well plate into 40μL of RNAlater (AM7021, Ambion). The cells in RNAlater were mixed with 40μL lysis buffer (3% Triton X-100, 100 mM DTT, RNAse inhibitor, H2O). Indexing Oligobeads (15μL) were added to the cell lysis mix and after 5 min incubation the beads were collected with a magnet and washed 2 times with 6× Saline-sodium citrate (SSC) (Invitrogen, 15557044) and once with 1× Maxima RT Buffer (Thermo Fisher, EP0743). Bead mixture was combined with reverse transcriptase (RT) mix (1 × Maxima RT buffer, Maxima H- RTase (Thermo Fisher, EP0743), RiboLock RNase inhibitor (Thermo Fisher, EO0382), 10 mM dNTPs (Thermo Fisher, R0193) and 50 μM Template Switch Oligo (5M Betaine Solution 15825238)). Samples were incubated for 30 min at 22°C and 90 min at 52°C. After the incubation, beads were washed with TE-SDS and TE-Tween. cDNA was amplified by PCR according to the protocol described in Macosko et al.^102^ The PCR products were pooled together in sets containing different Indexing Oligos and purified with Agencourt AMPure XP Beads (Beckman Coulter, A63881) according to the manufacturer’s instructions. The 3′-end cDNA fragments were prepared for sequencing using the Nextera XT tagmentation reaction (Illumina, FC-131-1096). The reaction was performed according to the manufacturer’s instructions, except for the P5 SMART primer that was used instead of the S5xx Nextera primer. Each set of samples that were pooled after the PCR reaction was tagmented with a different Nextera N7xx index (Illumina, FC-131-1001). Subsequently, the samples were PCR amplified and purified twice using Agencourt AMPure Beads. The libraries were sequenced on an Illumina NextSeq 500, with a custom primer producing read 1 of 21 bp and read 2 of 62 bp. Sequencing was performed at the Biomedicum Functional Genomics Unit of the Helsinki Institute of Life Science and Biocenter Finland at the University of Helsinki, Finland. The data is available at Gene Expression Omnibus database (GSE259326).

#### Isolation of primary intestinal fibroblasts

Primary intestinal fibroblasts were isolated from male and female mice using a modified version of a previously published protocol.^43^ The whole small intestine was washed with PBS, opened longitudinally, and cut into 1-2cm fragments. Tissue fragments were incubated in PBS containing 5 mM EDTA and 1 mM DTT for 20min and placed horizontally in a rotating incubator at 37°C and 250rpm. The EDTA solution was removed by washing the tissue pieces three times with ice-cold PBS by vigorously shaking for 15 s by hand, followed by foam removal and replacement of PBS between shakes. After the washes, the pieces were placed in PBS and minced thoroughly with a scalpel. The tissue pieces were placed in a collagenase digestion solution (2% fetal bovine serum in PBS with collagenase type II and IV; Gibco, 17101015; 17104019) and rotated at 250rpm horizontally for 20 min at 37°C. Cold fibroblast growth medium (DMEM containing 20% fetal bovine serum, 1% penicillin-streptomycin, and 1% L-glutamine) was added to stop the collagenase activity. Single cells were released from the digestion suspension by trituration, and the cells were pelleted by centrifugation for 10 min at 300 *g* and resuspended in 37°C growth medium. The suspension was plated on a Petri dish and cells were allowed to attach for 1h at 37°C, after which the plate was washed three times with PBS to remove debris and unattached cells, and fresh medium was added to the culture. For *in vitro* experiments, approximately 150,000 primary fibroblasts were plated on six-well plates and allowed to attach overnight before starting the treatments indicated in the text. For each biological replicate, at least two technical replicates per treatment were included. Lipopolysaccharide (LPS) (L4516, Sigma-Aldrich) was used at 1000 ng/mL.

#### Staining of intestinal organoids

Organoids were grown in 8 chamber slides (354108, Corning) and fixed with 4% paraformaldehyde in PBS for 1 h in RT. After washing with PBS, the organoids were permeabilized with 0.5% Triton-X-PBS for 30 min in RT and blocked with 10% of normal goat serum in 0.25% Triton-X-PBS for 60 min. Primary antibodies anti-GFP (ab290, Abcam, 1:500), Pyy (bs-2265r, Bioss, 1:100), serotonin (ab66047, Abcam, 1:500) were diluted in the blocking buffer and incubated for an hour in RT and washed once with 0.25% X-100-PBS followed by three PBS washes. The nuclei were stained with 10 μg/mL of Hoechst in PBS (33342, Thermo Fisher) for 5 min and the slides were mounted with Immu-Mount (9990402, Fisher Scientific). The secondary antibody controls were included for all experiments (Figure S10A).

#### Quantification of 5-HT+ and PYY+ cells in organoids

Mouse intestinal organoids from male mice were cultured as described above ENR media with or without GDNF and GFRA1 (100 ng/mL) for two days followed by culture in basal media or basal media supplemented with 100 ng/mL GDNF and GFRA1 for another two days. Organoids were fixed and stained as described above. Counting of the 5-HT+ and PYY+ cells per organoid was done using a Zeiss Axio Imager M2 microscope. The area of each organoid was measured with ImageJ and the number of positive cells was normalized to the organoid area in pixels. The relative fold change of 5-HT+ and PYY+ cells per organoid to the average of control organoids is shown.

#### Gene editing using CRISPR-Cas9

The gRNA targeting mouse *Ret* locus was designed using Benchling Software. (5′CAATCTTGCGGCTGTCACGG3′ targeting exon 14) and cloned into LentiCRISPRv2 plasmid (Addgene #52961, a gift from Feng Zhang) as described.^103^^,^^104^ For lentivirus production, 6 million HEK293FT cells were seeded and transfected after 24 h with the LentiCRISPRV2-Retg1 construct together with the pLp Δ8,9 packaging plasmid and VSVG envelope plasmid using lipofectamine 2000 transfection reagent (Invitrogen, 11668019) and Opti-MEM reduced serum medium (Gibco, 31985-047). Viruses were collected after 48 h, filtered through a 0.22 μm filter, and stored at −80°C until use. Intestinal crypts from wild-type male mouse were isolated as described above and lentiviral transduction was performed as reported^105^ with small modifications. Briefly, after the crypt isolation, the crypts were plated in 20-μL domes containing 75% Matrigel (356231; Corning) and 25% organoid growth media with ENR. The domes were overlaid with ENR media, which was changed every second day. To enrich for stem cells, the growth medium was supplemented with 3 μM CHIR99021 (SML1046; Sigma) and 1 μM valproic acid (PHR1061; Sigma) (ENR-CV medium).^106^ The enrichment was continued for 5 days, with the medium changed every second day. The organoids were harvested by incubating them in an organoid harvesting medium on ice for 30 min and dissociated into single cells in TrypLE Express (12605010; Thermo Fisher) containing 10% DNase1 (1014159001; Roche) for 1 min at 37°C, followed by trituration on ice. The dissociated cells were suspended in ENR-CV medium supplemented with 10 μM Y27632 and 8 μg/mL polybrene. The suspension was combined with an equal volume of lentivirus and spinoculated at 600g for 1 h, followed by a 4-h incubation at 37°C, collected and washed with phosphate-buffered saline (PBS), and plated in Matrigel, as above. The transduced organoids were selected with 1 μg/mL puromycin (A1113803, Gibco) for 10 days (until 2 rounds of control organoids were dead) and the surviving organoids were expanded in ENR-CV media. The organoids were passaged by harvesting as above and by trituration with a 200-μL pipette tip before embedding into Matrigel. The success of Ret targeting was verified using quantitative PCR and Sanger sequencing, and quantified with Inference of CRISPR Edits (ICE) software (Synthego).

#### Tissue preparation and staining

Small intestinal and colon tissues were collected, washed with PBS, and fixed in 4% paraformaldehyde (PFA) at +4°C for 24 h. During the fixation, PFA was replaced once. The intestines were embedded in paraffin as Swiss rolls with Sakura Tissue-Tek VIP 5 jr. tissue processing system. 5 μm thick sections were cut and deparaffinized, and antigen retrieval was performed using 1× Dako antigen retrieval solution either pH 6 or pH 9 (S1699 & S2367, Agilent) in +95°C for 20 min followed by 30 min in room temperature (RT). Blocking was performed using TNB blocking buffer (0.1M TRIS-HCl, 0.15M NaCl pH 7.5, 0.5% TSA Blocking Reagent (FP1012, PerkinElmer)) for 30 min in RT. Primary antibodies anti-GFP (ab290, Abcam, 1:500 dilution) and anti-Pdgfra (AF1062, R&D systems, 1:100 dilution), Anti-RFP (600-401-379, Rockland, 1:400), ChgA (ab45179, Abcam, 1:200), PYY (bs-2265r, Bioss, 1:100), 5-HT (ab66047, Abcam, 1:500), E-cadherin (U3254, Sigma, 1:100) were incubated overnight at +4°C. Secondary antibodies (Alexa Fluor 488 donkey anti-rabbit (A21206) and Alexa Fluor 594 donkey anti-goat (A11058)) were incubated in blocking buffer (1:500 dilution) for 1 h in RT and washed 3 × 5 min in 0.1% TBS-Tween. The nuclei were stained with 10 μg/mL Hoechst 33324 (62249, Thermo Fisher Scientific) in PBS for 3 min. Slides were rinsed with TBS and water before mounting with Immu-Mount (9990402, Fisher Scientific). The secondary antibody controls were included for all experiments (Figure S10B).

#### Immunohistochemistry for enteric nerves

The small intestine comprising proximal duodenum and distal ileum were fixed in 4% PFA at 4°C for 8 h. Longitudinal muscle/myenteric plexus preparations (LMMP) from proximal duodenum and distal ileum were used for immunohistochemical analysis. The LMMPs were peeled off from fixed tissue under a dissection microscope. The LMMPs were washed three times for 20 min per wash to discard residual PFA. LMMPs were permeabilized with 0.2% Triton X-100 (Thermo Fisher Scientific) in PBS for 2 h at RT. Then, the LMMPs were blocked in blocking buffer (5% normal donkey serum (Abcam), 5% BSA (Thermo Fisher Scientific), 0.2% Triton X-) followed by incubation with rabbit anti-beta III tubulin (Tuj1, 1:1000, Abcam ab18207) diluted in blocking buffer overnight at 4°C. After washing three times in PBS (20 min per wash), the LMMPs were incubated for 2 h at RT with donkey anti-rabbit Alexa 488 (1:500, Abcam, Ab150065) diluted in blocking buffer. Finally, the LMMPs were washed three times in PBS (20 min per wash), and a coverslip was placed on the positively charged slide (Thermo Fischer Scientific, Menzel-Gläser Superfrost plus) using mounting media (Thermo Fischer Scientific, Shandon Immu-Mount). Slides were imaged with a Zeiss Axio Imager (Oberkochen, Germany) microscope.

#### Imaging and image analysis

Imaging was performed with a Leica TCS SP8 CARS Confocal microscope and with Zeiss Axio Imager M2 microscopes and scanned using a 3DHISTECH Pannoramic 250 FLASH III digital slide scanner. Manual counting of PYY+ and 5-HT+ cells from the intestinal tissue was done from scanned images using CaseViewer. The number of positive cells in a field of view was counted. For quantification, 20× magnification was used for the duodenum and 30× magnification for the ileum. Data scoring and analysis were performed blinded to the genotype of the mice. Total epithelial area was measured using ImageJ by adjusting the threshold to cover the whole epithelia for each image. Villus height and crypt depth was measured manually with ImageJ. Tuj1+ area counts (%) were performed with ImageJ. Images were turned into binary images and the area covered by Tuj1+ fibers were quantified using the Analyze Particles function.

#### Quantitative PCR

RNA from organoids and primary fibroblasts was isolated using the Nucleospin RNA Plus isolation kit (740984.250 Macherey-Nagel) according to the manufacturer’s recommendations. Complementary DNA was produced with TaqMan reverse transcription kit (N8080234, Thermo Fisher Scientific) according to the protocol except that the final concentration of MgCl was 5 mM and the final concentration of MultiScribe reverse transcriptase was 1 U/μL. Reverse transcription reaction was done with 2720 Thermal Cycler (Applied Biosystems). qPRC was performed with KAPA SYBR FAST qPCR Master Mix Universal reagent (KK4617, KAPA Biosystems). All primers were diluted in nuclease-free water to a final concentration of 500 nM with a reaction volume of 16 μL. Samples were run with StepOnePlus Real-Time PCR System (Thermo Fisher Scientific). Actb was used as the internal control. The relative fold change was calculated using the delta-delta C_T_ method.^107^ List of used primers is in Table S1.

#### Correlation of *GDNF* mRNA levels with EEC signatures in the human colon

Raw RNA-sequencing read counts for the healthy intestinal samples from the Cancer Genome Atlas colon cancer cohort (TCGA-COAD) were uploaded through the Genomic Data Commons (GDC) data portal.^57^ To estimate the per sample enrichment score for different intestinal epithelial cell types, gene set variation analysis (GSVA) was performed for individual samples (*n* = 51) using the unfiltered table of raw read counts as an input.^108^ Gene sets for human intestinal epithelial cell types were previously defined.^58^ The Spearman-correlation coefficients and statistical significance between *GDNF* RNA expression (logCPM) and cell type enrichment scores were calculated using the R-package cor.test. *GDNF* gene itself was not included in any of the epithelial gene sets. The entire dataset included 51 samples and all of them were allocated to the experiment.

#### RNA sequencing and analysis

GDNF-treated organoids were harvested from two female mice (experiment 1 and 2) and one male mouse (experiment 3, P2_metadata file at Gene Expression Omnibus database GSE259326). The 3′ RNA sequencing was performed by the Biomedicum Functional Genomics Unit at the Helsinki Institute of Life Science and Biocenter Finland at the University of Helsinki. The 3′ RNAseq is based on the Dropseq method for single-cell sequencing^102^ and involves priming mRNA with an oligo dT primer containing a 12 bp barcode and an 8 bp UMI sequence which can be used to remove PCR duplicates during data analysis. Single-stranded cDNA is then converted into double-stranded cDNA using the template switch effect and the double-stranded cDNA product is PCR amplified with another set of primers (SMART PCR primer). Samples were pooled and PCR sequencing pools were generated with Nextera i7 primers and the Dropseq P5 primer. Sequencing was performed on the NextSeq High Output 75 cycle flow cell on the NextSeq 500. Quality library metrics were assessed using multiqc tool.^109^ The fastq files were aligned to the mouse reference genome (GRCm38) using STAR-aligner (v2.7.8a).^110^ Samples were demultiplexed and a count matrix of unique molecular identifier (UMI) counts was created using the *CreateDGEMatrix* command from the suite of tools BRB-seqToolsv1.4 (https://github.com/DeplanckeLab/BRB-seqTools). R2 aligned BAM and R1 fastq files were used as input. The R1 read had a length of 21 nucleotides: 12 nucleotide sample barcodes, 8 nucleotide UMI, and one extra nucleotide. Differential expression analysis was performed with the Deseq2 package^111^ following the Deseq2 pipeline (http://bioconductor.org/packages/devel/bioc/vignettes/DESeq2/inst/doc/DESeq2.html). In the *DESeqDataSetFromMatrix* function, the design matrix included experiment and treatment for cultured organoids and GFP for sorted single cells. Data were pre-filtered to keep only rows that have at least 5 read counts. All the samples in the results table with *BaseMean* value less than 1 were excluded as well as samples without any p-adjusted (*padj)* value. *padj* value of <0.1 was used for significant genes. Data transformation and visualization were done using variance stabilizing transformations.^112^ Computational resources for all the data processing were provided by CSC – IT Center for Science, Finland. Raw fastq files, barcode sample information as well as processed UMI count matrix are available at Gene Expression Omnibus database GSE259326.

#### Gene set enrichment analysis

Gene set enrichment analysis (GSEA)^101^^,^^113^ was performed using the Hallmark dataset,^114^ intestinal epithelial cell type markers gene sets^49^ and EEC progenitor gene sets.^55^ The analyses were conducted using the GSEA v4 software.^101^ Normalized enrichment scores (NES) and false discovery rates (FDR q-value) are displayed.

#### scRNA-seq data analysis

scRNA-Seq data of murine small intestinal fibroblasts^13^ (GSE130681, sample GSM3747599), colonic mesenchymal cells^44^ (GSE142431, samples GSM4227211-GSM4227215) and small intestinal epithelial cells^49^ (GSE92332) were acquired from the Gene Expression Omnibus and re-analyzed in R using the Seurat v4 pipeline.^100^ Genes detected in fewer than three cells were initially filtered out as were cells that expressed less than 200 genes. Further prefiltering was done by using the following quality control metrics to exclude possible doublets and cells expressing high levels of mitochondrial or ribosomal RNA. For GSE130681: cells containing more than 10% mitochondrial or 25% ribosomal counts were filtered out. Cells with fewer than 500 or more than 3000 unique feature counts (nFeature) and cells with fewer than 1000 or more than 8000 unique molecules detected (nCount) were filtered out. For GSE142431: cells containing more than 20% mitochondrial or 30% ribosomal counts, cells with fewer than 250 or more than 1500 nFeature counts, and cells with fewer than 250 or more than 4000 nCount detections were filtered out. For GSE92332: cells containing more than 20% ribosomal counts, cells with fewer than 500 or more than 2700 nFeature counts, and cells with fewer than 200 or more than 11000 nCount detections were filtered out. The data were normalized (SCTransform), integrated, and clustered following the Seurat v4 integration pipeline for SCTransformed data (https://satijalab.org/seurat/archive/v4.3/integration_introduction). Major cell populations were identified by cross-referencing cluster-specific gene expression with established marker genes of different cell types.

#### ScRNA-seq data analysis of murine EECs

The data^55^ (GSE113561) were acquired from the Gene Expression Omnibus and re-analyzed in R using the Seurat v4 pipeline.^100^ From raw data, only the sorted cells in 96-well and 384-well plates were used for analysis. Prefiltering was done to each plate separately to exclude possible doublets as well as cells expressing high levels of mitochondrial or ribosomal RNA. Genes detected in fewer than three cells were initially filtered out as were cells that expressed less than 200 genes. Further prefiltering was done using the following quality control metrics: cells containing more than 15% mitochondrial or 20% ribosomal counts, cells with fewer than 200 or more than 10000 unique feature counts, and cells with fewer than 500 or more than 50000 unique molecules detected were filtered out. All the ERCC spike-in genes were filtered out from the data as in the original publication. Each plate was normalized (SCTransform) and mitochondrial genes were regressed out using *vars.to.regress* command in the normalization. Separate plates were integrated with *k.weight = 72* and clustered following the Seurat v4 integration pipeline for SCTransformed data (https://satijalab.org/seurat/archive/v4.3/integration_introduction). Cells with >6 *Kcnq1ot1* and *Rn45s* indicating clustering artifacts were excluded from the analysis as was a cluster with high *Lgr5* expression indicating a stem cell population. A cluster with high expression of mitochondrial genes was excluded from the final analysis. Major cell populations were identified by cross-referencing cluster-specific gene expression with established marker genes of different cell types.

#### Analysis of scRNA-Seq data of human cells

The data from human colon mesenchyme^59^ (GSE114374, samples GSM3140593 and GSM3140594) and human intestinal organoids^10^ (GSE146799) were acquired from the Gene Expression Omnibus and re-analyzed in R using the Seurat v4 pipeline.^100^ Genes detected in fewer than three cells were initially filtered out as were cells that expressed less than 200 genes. Further prefiltering was done by using the following quality control metrics to exclude possible doublets and cells of bad quality. For GSE114374: cells containing more than 2.5% mitochondrial or 15% ribosomal counts were filtered out. Cells with fewer than 500 or more than 3000 unique feature counts (nFeature) and cells with fewer than 1500 or more than 3500 unique molecules detected (nCount) were filtered out. For GSE146799: cells containing more than 35% or less than 1% mitochondrial or more than 20% ribosomal counts were filtered out. In addition, cells with fewer than 2000 or more than 7000 nFeature counts, and cells with fewer than 500 or more than 50000 nCount detections were filtered out. The data were normalized (SCTransform) while regressing out mitochondrial and ribosomal gene expression and clustered following the Seurat v4 pipeline for SCTransformed data (https://satijalab.org/seurat/archive/v4.3/integration_introduction). Major cell populations were identified by cross-referencing cluster-specific gene expression with established marker genes of different cell types.

### Quantification and statistical analysis

The graphs were drawn, and statistical analyses were performed using the Prism 9 software. Non-paired T-test and ONE-WAY ANOVA were used as statistical tests as detailed in the figure legends. The statistical details of the experiment can be found in figure legends or in the method section. Asterisks denote statistical significance (*p* < 0.05 unless otherwise specified). Mean and standard deviation are shown in bar plots.
