# Supplementary material for: Mesenchymal GDNF promotes intestinal enterochromaffin cell differentiation
Source: iScience. 2024 Oct 24;27(12):111246. doi: 10.1016/j.isci.2024.111246 (PMC11616604; doi:10.1016/j.isci.2024.111246)
Supplement: Document S1. Figures S1–S10 and Table S1 [file mmc1.pdf]

iScience, Volume *27*

Supplemental information

Mesenchymal GDNF promotes intestinal enterochromaffin cell differentiation

Toni T. Lemmetyinen, Emma W. Viitala, Linnea Wartiovaara, Pekka Päivinen, Heikki T. Virtanen, Nalle Pentinmikko, Pekka Katajisto, Tomi P. Mäkelä, Timothy C. Wang, Jaan-Olle Andressoo, and Saara Ollila

Supplemental information

Figure S1

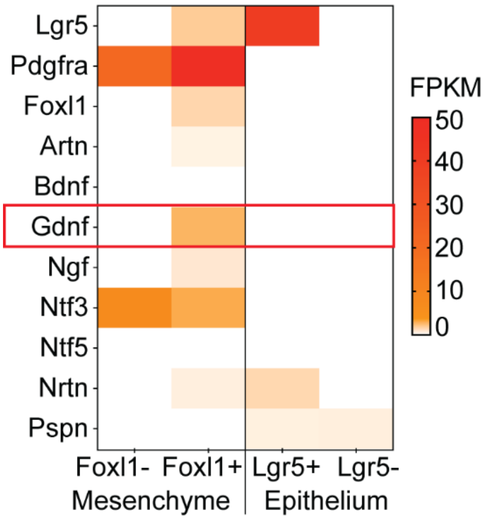

Figure S1. Neurotrophic factor expression in intestinal mesenchymal and epithelial cells. Related to Figure 1. RNAseq-analysis of indicated genes from sorted Foxl1+ and Foxl1- mouse intestinal mesenchymal cells and sorted Lgr5+ and Lgr5- cells from mouse intestinal epithelium[S1] (GSE94072). FPKM, fragments per kilobase of transcript per million mapped reads. *Gdnf* is exclusively expressed in the *Foxl1*-expressing, *Pdgfra*^{high} mesenchymal cells.

Figure S2

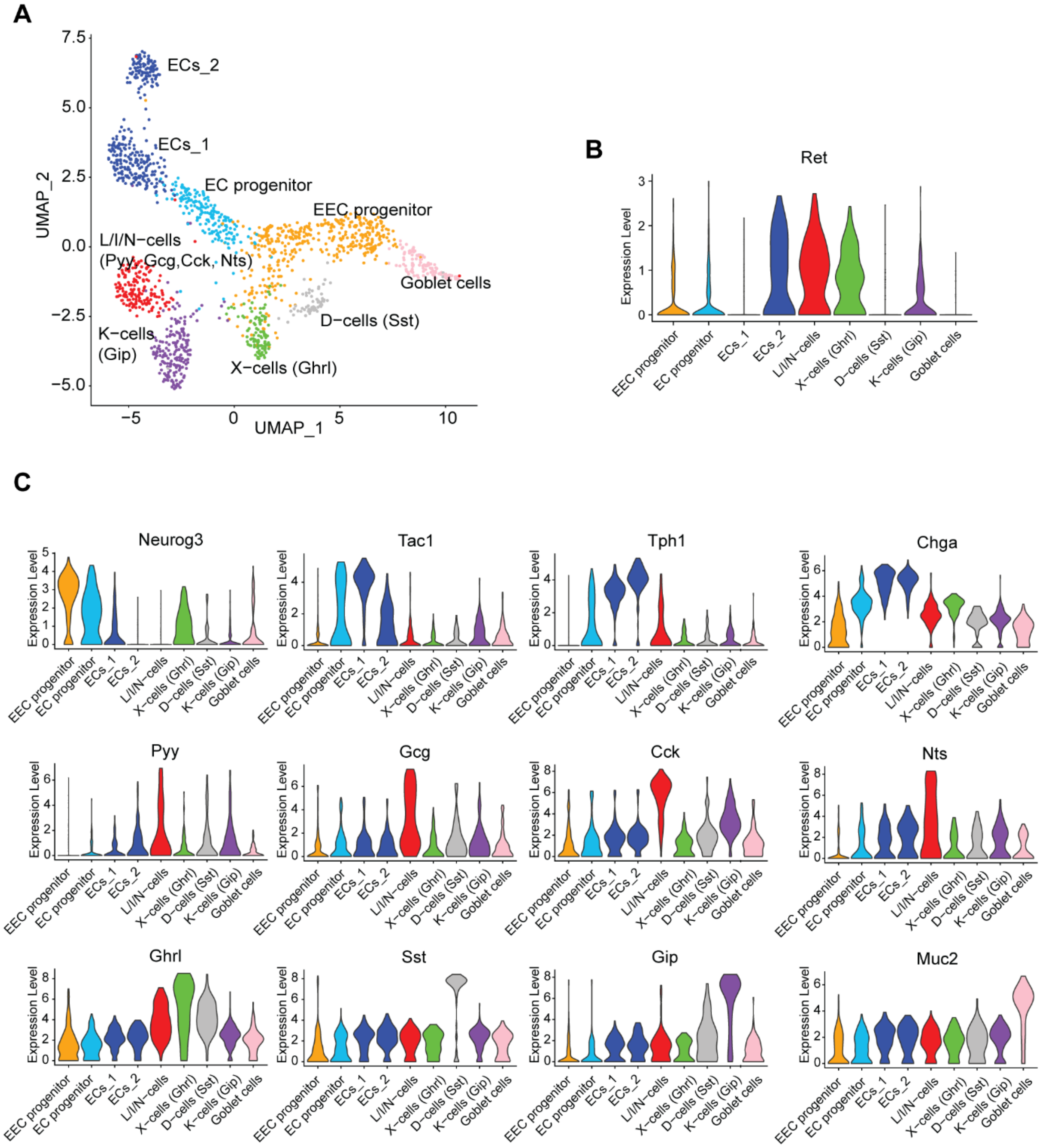

Figure **S2.** **Expression of RET in EEC subtypes. Related to Figure 3.** (A) UMAP of EEC subtype populations[S2] (GSE113561). (B) Violin plot of *Ret* expression in the indicated clusters. (C) Violin plot of indicated genes depicting known markers for each EEC subtype.

Figure S3

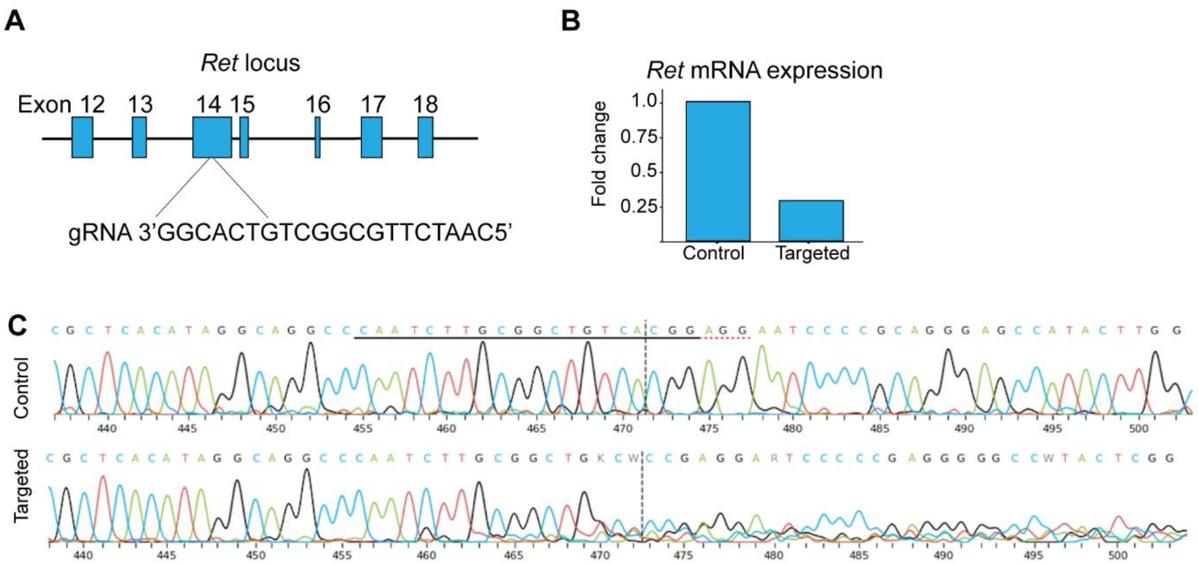

Figure **S3****. Generation of *Ret* knock-out organoids using CRISPR-Cas9. Related to Figure 3.** (A) A gRNA was designed to target the kinase domain in exon 14 of the *Ret* locus. (B) Relative expression of *Ret* mRNA in control and targeted organoids. (C) Sanger sequencing reveals a loss of wild-type sequence at the gRNA binding site in targeted organoids. The black line represents the gRNA binding site, the dashed red line represents the PAM site. Analysis powered by Synthego.

Figure S4

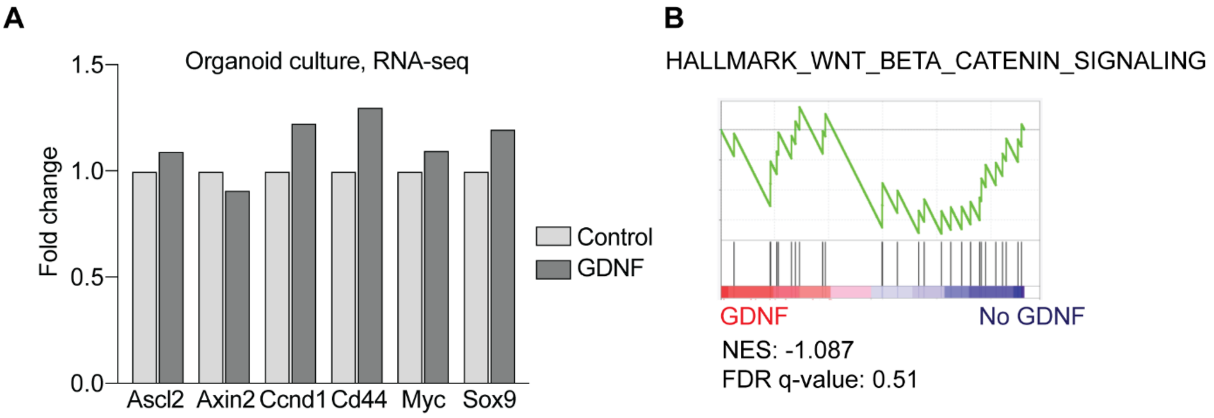

Figure S4. Wnt signaling is not induced by GDNF in intestinal organoids. Related to Figure 3. (A) Bulk RNA-seq results of indicated Wnt target genes from GDNF-treated intestinal organoids compared to control (No GDNF) after 4 days of culture. None of the changes were significantly different. (B) GSEA plot of GDNF-treated organoids against the Hallmark signature "Wnt-beta-catenin signaling" from the Molecular Signatures Database (MSigDB)[S3].

Figure S5

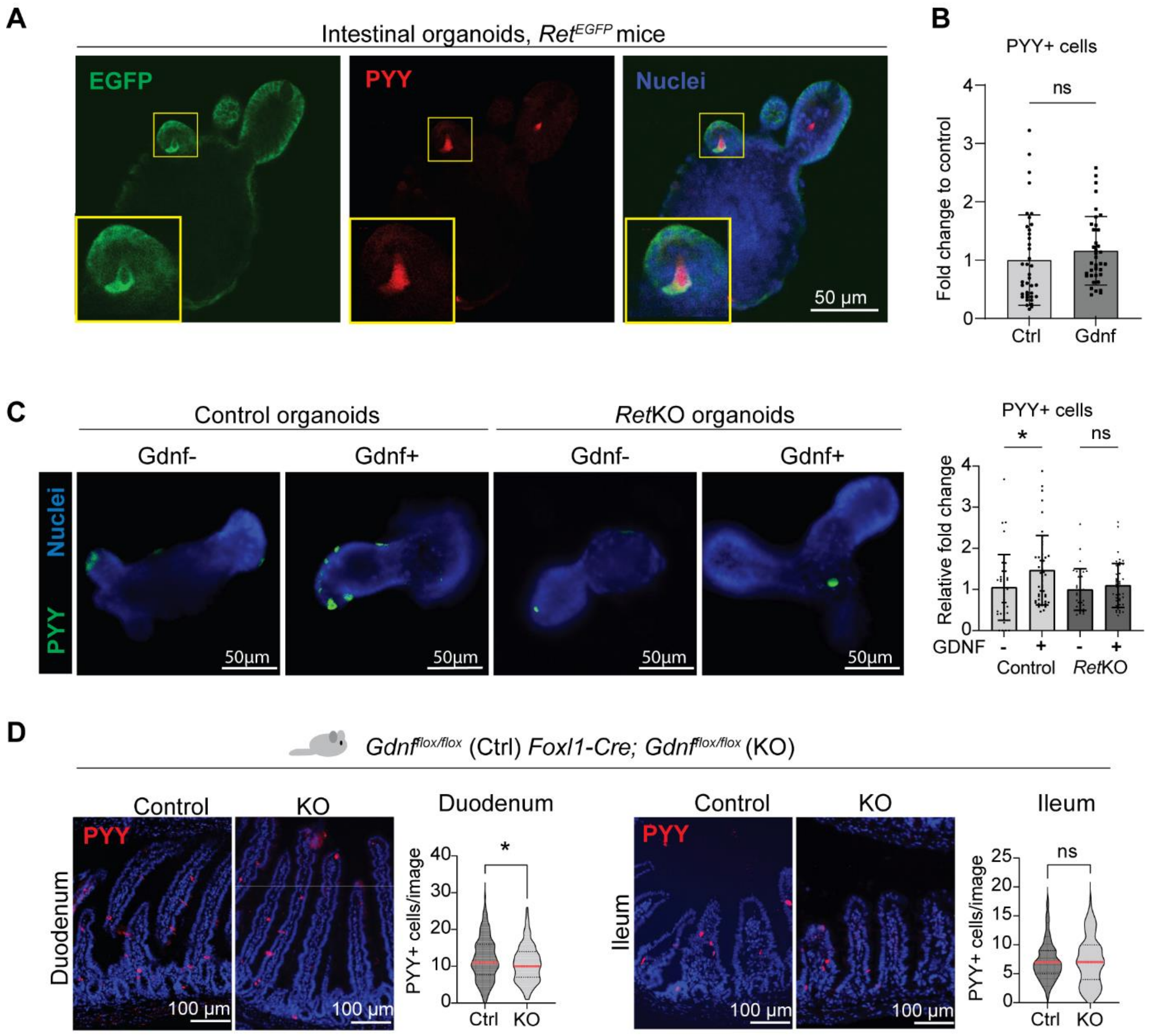

Figure S5. Quantification of PYY+ cell frequency induced by GDNF. Related to Figure 5. (A) Immunofluorescence staining of EGFP (RET) and PYY in mouse intestinal organoids. (B) Quantification of PYY+ cells in organoids derived from WT male mice after 4 days of GDNF treatment as compared to control organoids. Relative fold change is shown. N = 39 control organoids and 37 GDNF-treated organoids were counted from 3 independent mice. Asterisks indicate statistical significance (*p<0.05, two-tailed unpaired t-test). Mean and standard deviation are shown. (C) Representative images and quantification of PYY+ cells on control and *Ret*KO organoids with or without GDNF treatment. Control n=38, Control+GDNF n=49, *Ret*KO control n=34, *Ret*KO+GDNF n=42 organoids. (*p<0.05, one-way ANOVA with Tukey's post hoc test). Mean and standard deviation are shown. (D) Representative images and quantification of PYY positive cells in duodenum and ileum of

control and KO mice. PYY+ cells in duodenum counted from at least 178 20X images from at least six individual mice. Duodenum: n=178 images from n=7 control mice, n=189 images from n=6 KO mice p-value. Ileum: PYY+ cells in ileum counted from at least 135 30X images. Control, n=164 images; KO, n=135 images from n=7 mice. Red line in the graph indicates the mean value and the dashed lines indicate standard deviation. Asterisks indicate statistical significance (*$p<0.05$, two-tailed unpaired t-test).

Figure S6

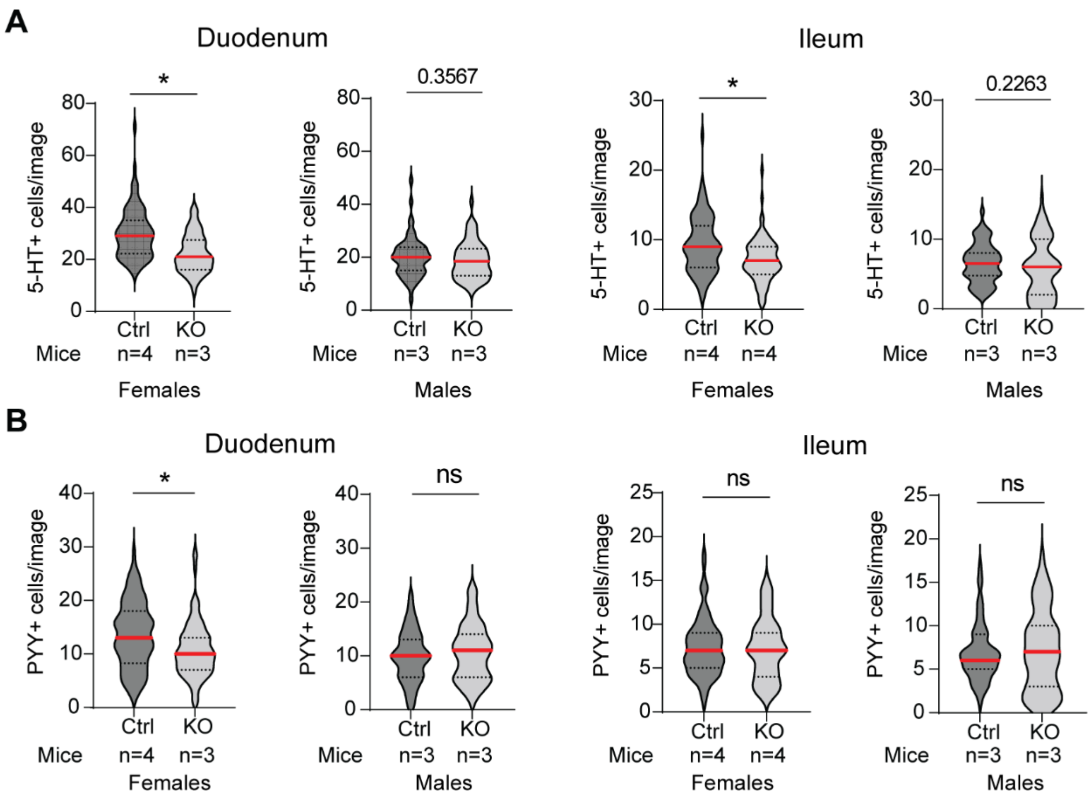

Figure S6. 5-HT+ and PYY+ cell counts in female and male Foxl1-Cre;Gdnf$^{flox/flox}$ mice. Related to Figure 5. (A) Data of 5-HT+ cells in duodenum and ileum (Figure 5E) shown separately for male and female mice. Duodenum females: n=88 images from n=4 control mice, n=49 images from n=3 KO mice. Duodenum males: control, n=60 images; KO, n=62 images from n=3 mice. Ileum females: control, n=85 images; KO, n=89 images from n=4 mice. Ileum males: control, n=62 images; KO, n=83 images from n=3 mice. Red line in the graph indicates the mean value and dashed lines the standard deviation. Asterisks indicate statistical significance (*p<0.05, two-tailed unpaired t-test). (B) Data of PYY+ cells in duodenum and ileum (Figure S5D) shown separately for male and female mice. Duodenum females: control, n=92 images from n=4 mice; KO, n=92 images from n=3 mice. Duodenum males: control, n=86 images; KO, n=97 images from n=3 mice. Ileum females: control, n=86 images; KO, n=63 from n=4 mice. Ileum males: control, n=78 images; KO, n=72 images from n=3 mice. Red line in the graph indicates the mean and dashed lines the standard deviation. Asterisks indicate statistical significance (*p<0.05, two-tailed unpaired t-test).

Figure S7

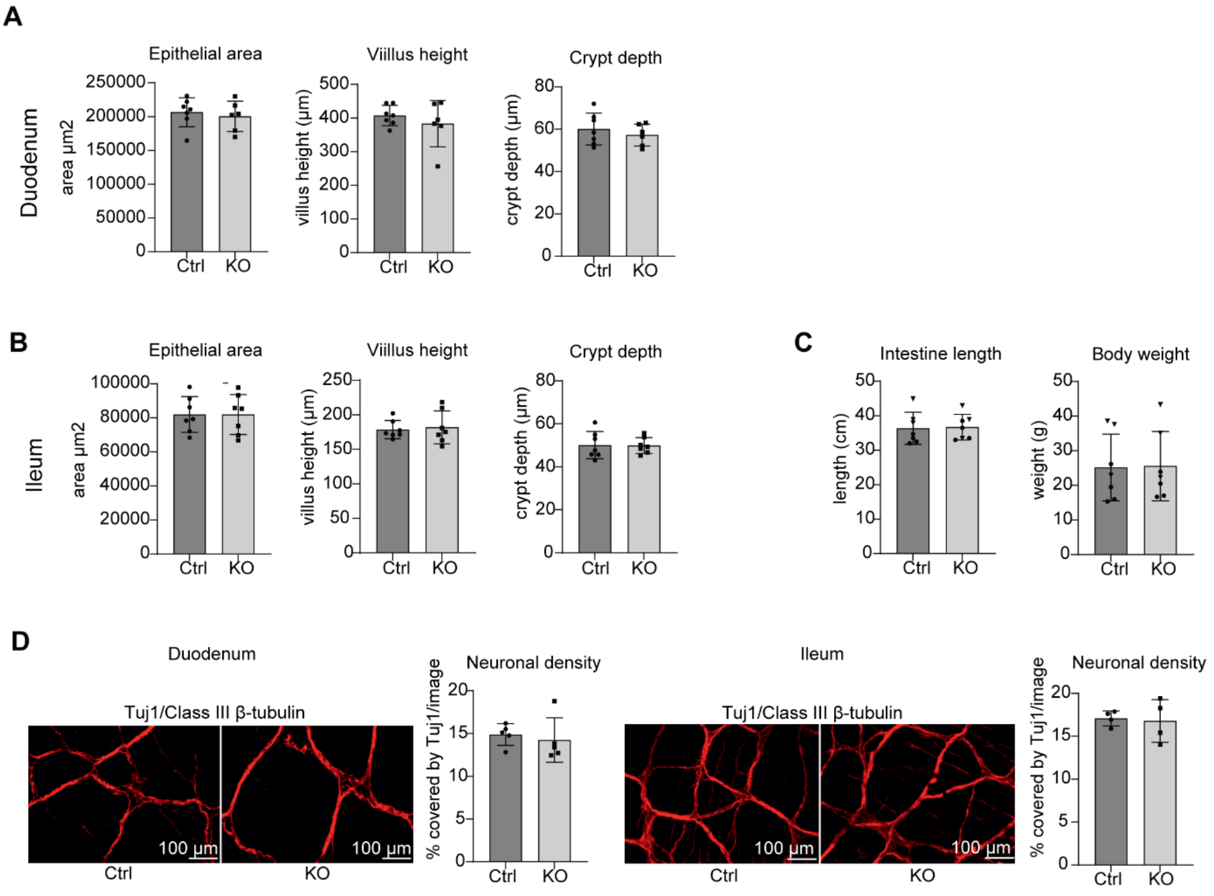

Figure S7. Characterization of Foxl1-Cre;Gdnf$^{flox/flox}$ mouse intestines. Related to Figure 5. (A-B) Average of total epithelial area (left graph), villus height (middle) and crypt depth (right) calculated from duodenum (A) and ileum (B) of *Foxl1-Cre* (Ctrl) mice (n=7) and *Foxl1-Cre;Gdnf$^{flox/flox}$* (KO) mice (n=6). Each dot represents the average value counted from at least 5 images in each mouse. Mean and standard deviation are shown. (C) Body weight and intestine length for *Foxl1-Cre* (Ctrl) mice (n=7) and *Foxl1-Cre;Gdnf$^{flox/flox}$* (KO) mice (n=7). Circles depict female and triangles male mice. Mean and standard deviation are shown. (D) Representative images and quantification of neuronal density in duodenum and ileum myenteric plexi of *Foxl1-Cre* (Ctrl) and *Foxl1-Cre;Gdnf$^{flox/flox}$* (KO) mice. At least 5 images were analyzed from each mouse. Duodenum: Ctrl n=5 mice, KO n=5 mice, Ileum: Ctrl n=4, KO n=4. Mean and standard deviation are shown. Asterisks indicate statistical significance (*p<0.05, two-tailed unpaired t-test).

Figure S8

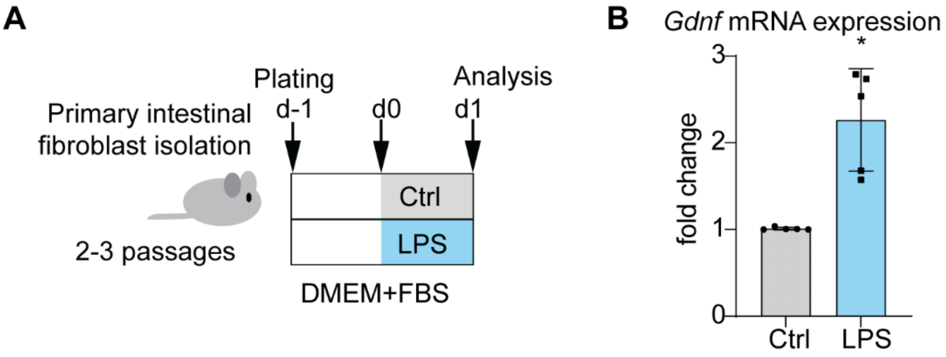

Figure S8. LPS induces *Gdnf* expression in primary intestinal fibroblasts. Related to Figure 5. (A) Outline of the experiment. (B) Expression of *Gdnf* mRNA in LPS-treated primary intestinal fibroblasts as compared to untreated control fibroblasts. Each dot represents an independent primary fibroblast pool (n=5). Asterisk (*) indicates p<0.05 (two-tailed unpaired t-test). Mean and standard deviation is shown.

Figure S9

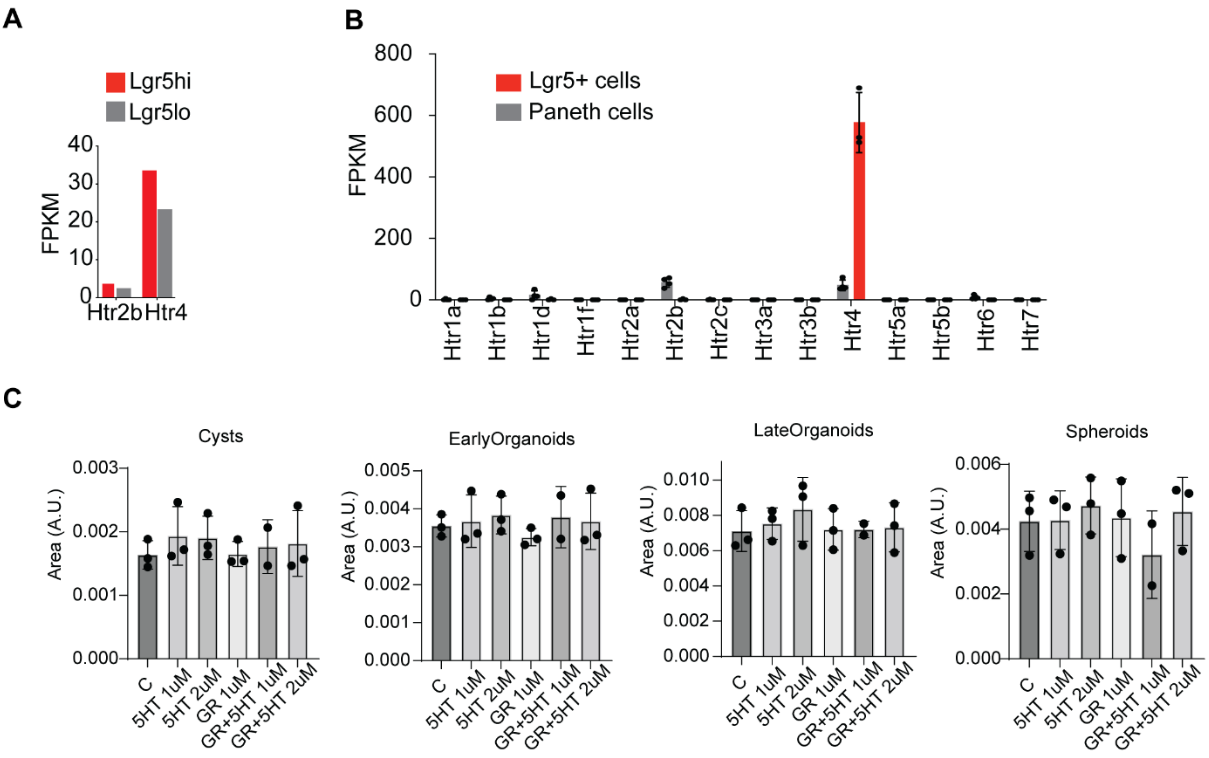

Figure S9. Expression of *Htr4* in Lgr5+ ISCs. Related to Figure 6. (A) *Htr2b* and *Htr4* were the only 5-HT receptors identified in RNA-sequencing of Lgr5-EGFP high (Lgr5hi) and Lgr5-EGFP low (Lgr5lo) cells[S4] (E-MTAB-6501). (B) Expression of all 5-HT receptors in sorted Lgr5+ cells and Paneth cells[S5] (E-MTAB-7916). (C) Quantification of the organoid size in different organoid classes (Cysts, EarlyOrganoids, LateOrganoids and Spheroids). Quantification of the organoid formation was done using the organoid classifier tool Tellu[S6]. Graphs depict the average organoid size from each experiment (n=3, except for GR+5-HT 1µM n=2 experiments). 2 images per well were analyzed from each experiment, and 3 technical replicates (wells) were analyzed per experiment. A.U., Arbitrary unit. Mean and standard deviation are shown.

Figure S10

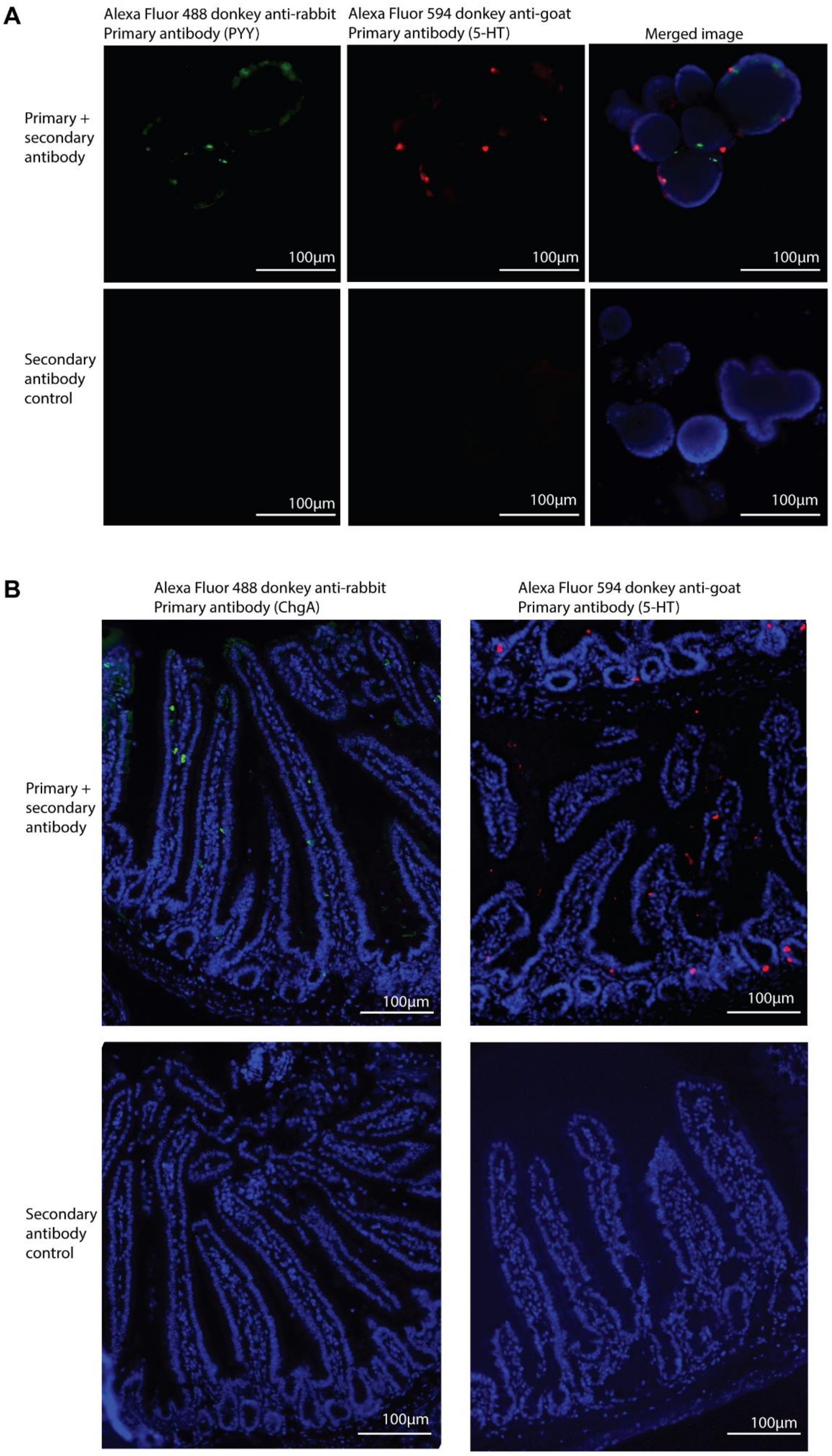

A
Alexa Fluor 488 donkey anti-rabbit
Primary antibody (PYY)
Alexa Fluor 594 donkey anti-goat
Primary antibody (5-HT)
Merged image
Primary + secondary antibody
Secondary antibody control
100μm
B
Alexa Fluor 488 donkey anti-rabbit
Primary antibody (ChgA)
Alexa Fluor 594 donkey anti-goat
Primary antibody (5-HT)
Primary + secondary antibody
Secondary antibody control
100μm

Figure S10. Secondary antibody controls for immunofluorescence stains. Related to STAR Methods. (A) Representative images of organoid stainings with Alexa Fluor 488 donkey anti-rabbit and Alexa Fluor 594 donkey anti-goat (top panel). Secondary antibody controls are shown in the bottom panel. (B) Representative images of tissue stainings with Alexa Fluor 488 donkey anti-rabbit and Alexa Fluor 594 donkey anti-goat (top panel). Secondary antibody controls are shown in the bottom panel.

Table S1. List of qPCR primers

Gene	Forward primer (5'-3')	Reverse primer (5'-3')
Actb	CTAAGGCCAACCGTGAAAAG	ACCAGAGGCATACAGGGACA
Alpi	AGGATCCATCTGTCCTTTGGT	TTCAGCTGCCTTCTTGTTCC
Cck	TGATTTCCCCATCCAAAGC	GCTTCTGCAGGGACTACCG
Gcg	TACACCTGTTCGCAGCTCAG	TTGCACCAGCATTATAAGCAA
Gdnf	CGCTGACCAGTGACTCCAATATGC	TGCCGCTTGTTTATCTGGTGACC
Ghrl	GCCATGCTGCTGATACTGAG	CCCAGAGGACAGAGGACAAG
Gip	GAGTTCCGATCCCATGCTAA	TGTGCCTCTTTGTCCTCCTT
Htr4	CTGTCCCCTGTTCAACCACAAC	CTGTGAGGTGACACCGACTCTC
Lgr5	TAAAGACGACGGCAACAGTG	GCCTTCAGGTCTTCCTCAAA
Nts	TGCTGACCATCTTCCAGCTC	GAATGTAGGGCCTTCTGGGT
Pyy	ACGGTCGCAATGCTGCTAAT	GCTGCGGGGACATCTCTTTTT
Ret	TCCCTTCCACATGGATTGA	ATCGGCTCTCGTGAGTGGTA
Sct	GACCCCAAGACACTCAGACG	TTTTCTGTGTCCTGCTCGCT
Sst	ACCGGGAAACAGGAACTGG	TTGCTGGGTTCGAGTTGGC
Tac1	CGAGGGGGCGGCTAATTAAA	AGCTCCTGCTTTGCGGTATT
Tph1	ACGTCGAAAGTATTTTGCGGA	ACGGTTCCCCAGGTCTTAATC

References

S1. Shoshkes-Carmel, M., Wang, Y.J., Wangensteen, K.J., Tóth, B., Kondo, A., Massasa, E.E., Itzkovitz, S., and Kaestner, K.H. (2018). Subepithelial telocytes are an important source of Wnts that supports intestinal crypts. Nature *557*, 242-246. 10.1038/s41586-018-0084-4.

S2. Gehart, H., van Es, J.H., Hamer, K., Beumer, J., Kretzschmar, K., Dekkers, J.F., Rios, A., and Clevers, H. (2019). Identification of Enteroendocrine Regulators by Real-Time Single-Cell Differentiation Mapping. Cell *176*, 1158-1173.e1116. 10.1016/j.cell.2018.12.029.

S3. Liberzon, A., Birger, C., Thorvaldsdóttir, H., Ghandi, M., Mesirov, J.P., and Tamayo, P. (2015). The Molecular Signatures Database (MSigDB) hallmark gene set collection. Cell Syst *1*, 417-425. 10.1016/j.cels.2015.12.004.

S4. Gao, Y., Yan, Y., Tripathi, S., Pentinmikko, N., Amaral, A., Päivinen, P., Domènech-Moreno, E., Andersson, S., Wong, I.P.L., Clevers, H., et al. (2020). LKB1 Represses ATOH1 via PDK4 and Energy Metabolism and Regulates Intestinal Stem Cell Fate. Gastroenterology *158*, 1389-1401.e1310. 10.1053/j.gastro.2019.12.033.

S5. Pentinmikko, N., Iqbal, S., Mana, M., Andersson, S., Cognetta, A.B., 3rd, Suciu, R.M., Roper, J., Luopajärvi, K., Markelin, E., Gopalakrishnan, S., et al. (2019).

Notum produced by Paneth cells attenuates regeneration of aged intestinal epithelium. Nature *571*, 398-402. 10.1038/s41586-019-1383-0.

S6. Domènech-Moreno, E., Brandt, A., Lemmetyinen, T.T., Wartiovaara, L., Mäkelä, T.P., and Ollila, S. (2023). Tellu - an object-detector algorithm for automatic classification of intestinal organoids. Dis Model Mech *16*. 10.1242/dmm.049756.
